# Ultrahigh‐Temperature‐Tolerance Lithium Metal Batteries Enabled by Molecular‐Level Polymer Configuration Design with Low‐Entropy‐Penalty Effect

**DOI:** 10.1002/advs.202507191

**Published:** 2025-08-16

**Authors:** Weiting Ma, Shunshun Zhao, Shuang Wan, Jiajun Gong, Sinian Yang, Yong Chen, Shimou Chen, Guoxiu Wang

**Affiliations:** ^1^ State Key Laboratory of Chemical Resource Engineering, Beijing Key Laboratory of Electrochemical Process and Technology of Materials Beijing University of Chemical Technology Beijing 100029 P. R. China; ^2^ Centre for Clean Energy Technology University of Technology Sydney Broadway Sydney NSW 2007 Australia

**Keywords:** lithium metal batteries, low‐entropy penalty, polymer electrolyte, ultrahigh‐temperature‐tolerance

## Abstract

Despite their immense potential for next‐generation energy storage, the practical implementation of temperature‐tolerant lithium metal batteries (LMBs) under extreme thermal conditions continues to face formidable challenges. In this study, an ultrahigh‐temperature‐tolerance polymer‐based electrolyte (UPE) prototype with a low‐entropy‐penalty effect is proposed. This electrolyte features a carefully engineered molecular configuration that enables stable operation of polymer‐based LMBs across a broad temperature range (25–150 °C). Comprehensive experimental and theoretical analyses confirm that the unique “ester‐ether‐fluorinated segment” architecture enables the formation of a robust coordination framework through Li⁺‐multivalent ether/ester interactions and effective Li^+^‐ether strong‐solvent‐cage decoupling. The resulting polymer electrolyte integrates reactive carboxyl groups, alkali‐metal‐soluble ether moieties, and fluorinated segments that provide inert yet efficient ion conduction pathways. This synergistic configuration achieves high ionic conductivity, significantly improved lithium‐ion transference numbers, and excellent interfacial compatibility with lithium metal. This work presents a molecular‐level polymer design framework, providing a compelling direction for the development of high‐performance, thermally stable lithium‐metal batteries.

## Graphical abstract

An innovative molecular‐level strategy is employed to engineer an advanced ultrahigh‐temperature‐tolerant polymer‐based electrolyte (UPE) through rational polymer structural design (up to 150 °C). By synergistically incorporating reactive carboxyl groups, ether electrolytes, and fluorinated molecular segments, Li metal pouch cells with UPE can achieve stable cycling under ultrahigh temperature conditions (120 °C).

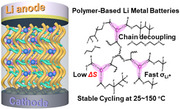

## 1 Introduction

**Figure 1:**
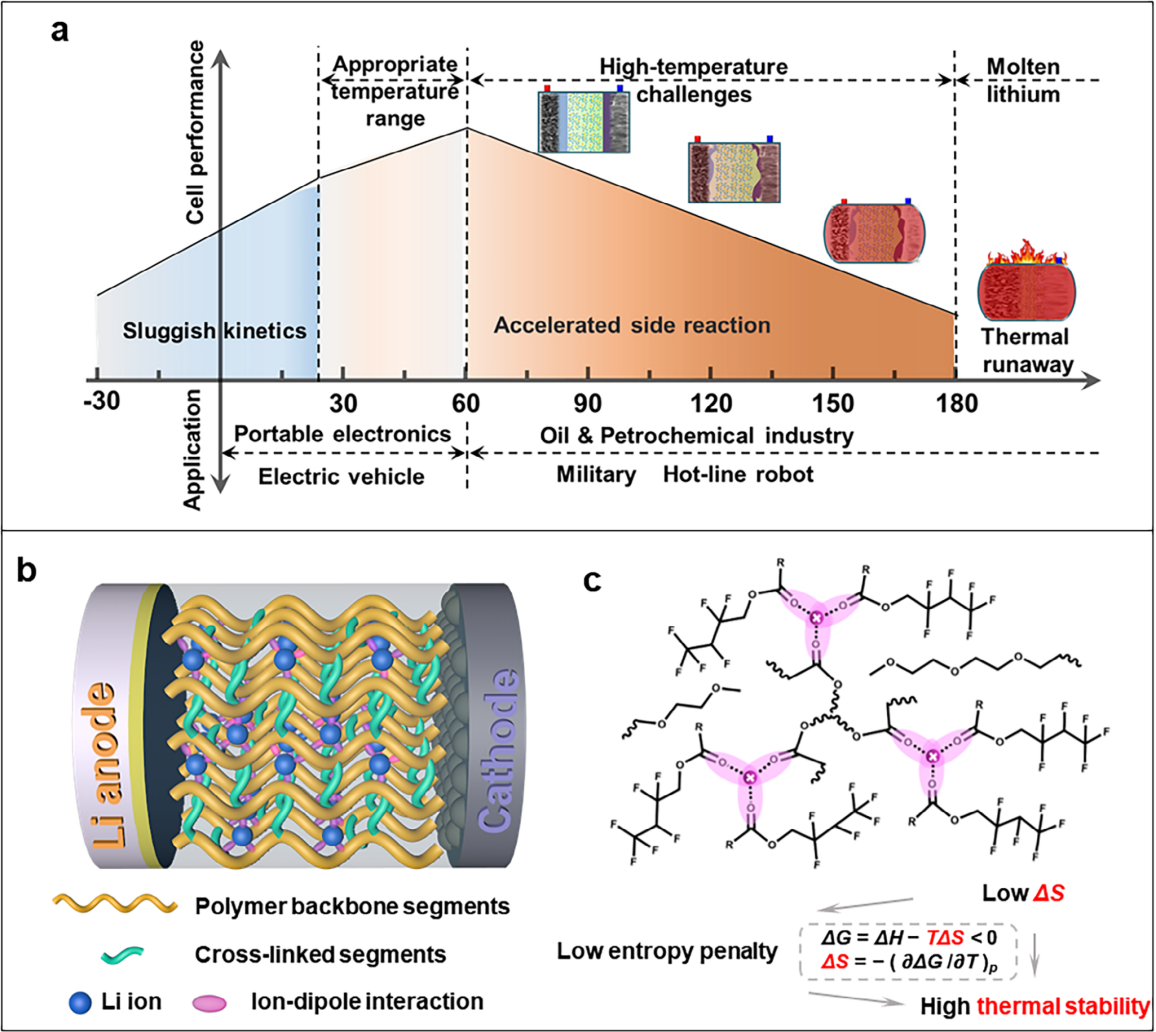
a) Challenges and applications of LMBs at different temperatures. b) Schematic illustration of UPE with a molecular‐level configuration design. c) Low entropy penalty internal molecular distribution with dynamic ion‐dipole cross‐linking mechanism.

Rechargeable lithium metal batteries (LMBs) with high‐temperature adaptability are experiencing increasing demand, driven by rapid advancements in fields like emergency rescue, healthcare, petrochemicals, and aerospace.^[^ ^1^, ^2^, ^3^, ^4^, ^5^, ^6^ ^]^ For example, medical‐grade lithium batteries used in sterilization equipment must demonstrate stable performance at temperatures exceeding 120 °C.^[^ ^3^ ^]^ However, developing and exploring LMBs for high and ultrahigh‐temperature environments (>100 °C) has received limited attention.^[^ ^7^ ^]^ Extensive research has predominantly focused on applications below 60 °C, particularly achieving optimal performance at room temperature or sub‐ambient conditions (**Figure** **1**a). This narrow focus has constrained advancements in stability and functionality at elevated temperatures, leaving a significant gap in addressing the demands of extreme thermal environments.^[^ ^2^, ^8^ ^]^

The intrinsic properties of battery components fundamentally determine the temperature resistance of LMBs. A widely observed phenomenon is that elevated temperatures raise the electronic energy levels of both the anode and cathode, inducing shifts in the highest occupied molecular orbital (HOMO) and lowest unoccupied molecular orbital (LUMO), which in turn narrow the energy gap between them.^[^ ^9^, ^10^ ^]^ The changes in energy levels exacerbate interfacial side reactions between electrodes and electrolytes, leading to cathode material degradation and lithium metal anode corrosion.^[^ ^11^ ^]^ These processes accelerate the decomposition of the solid electrolyte interface (SEI) and cathode electrolyte interface (CEI), ultimately resulting in rapid battery failure.^[^ ^12^, ^13^ ^]^ Consequently, LMBs suffer from pronounced capacity and power degradation at elevated temperatures, underscoring the critical need for electrolytes with enhanced thermal stability and performance. Among existing electrolyte types, ion transport in liquid electrolytes is typically governed by non‐covalent interactions between lithium ions and solvent molecules.^[^ ^14^ ^]^ Nonetheless, elevated temperatures significantly weaken the interactions between ions and solvent molecules, thereby destabilizing the solvation structure and exacerbating side reactions.^[^ ^15^ ^]^ Furthermore, commercial ester‐based liquid electrolytes are generally restricted to operating temperatures below 55 °C due to the unstable decomposition of lithium salts (LiPF_6_) and the high reactivity between ester groups (─C═O) and lithium metal.^[^ ^16^ ^]^ Elevated temperatures exacerbate the reaction between ─C═O and lithium metal, even when stable lithium salts such as LiTFSI or LiFSI are used.

Ether‐based electrolytes are another typical type of liquid electrolyte, widely recognized for their chemical compatibility with lithium metal.^[^ ^17^, ^18^ ^]^ However, studies have revealed that alkali metals can dissolve in these electrolytes, resulting in chemical instability and electronic leakage.^[^ ^19^ ^]^ Moreover, fluorinated solvent‐based localized high‐concentration and high‐concentration electrolytes can form robust inorganic‐rich SEI and CEI layers. Unfortunately, the fluorinated ether solvent participates in the ether‐solvent‐mediated dissolution of alkali metals. Yet, their performance under high temperatures remains insufficient for practical applications. Additionally, liquid lithium metal batteries face an inherent risk of separator shrinkage at elevated temperatures.^[^ ^20^ ^]^ Solid polymer and composite polymer electrolytes are two promising candidates for mitigating thermal runaway in LMBs, offering enhanced wide‐temperature stability and scalability for large‐scale production.^[^ ^21^, ^22^ ^]^ However, their operational temperature range is extremely narrow, primarily due to the thermal instability of the polymer backbones and inevitable residual solvents, and the loss of mechanical performance under elevated temperatures (above the glass transition temperature, T_g_).^[^ ^23^, ^24^ ^]^ Furthermore, these electrolytes with ultra‐dry configurations require elevated operating temperatures, and their performance under high‐temperature conditions (above 80 °C) remains uncertain.^[^ ^25^, ^26^ ^]^ While inorganic electrolytes exhibit fragile mechanical stability and liquid electrolytes present challenges for battery packaging technology at high temperatures, solid polymer electrolytes (SPEs) demonstrate significant development potential in ultra‐high‐temperature energy storage devices due to their design flexibility and modifiability. Consequently, the applicability of commercially available electrolytes in high‐temperature environments remains a subject of limited exploration and insufficient development.

Herein, an ultrahigh‐temperature‐tolerance polymer‐based electrolyte (UPE) prototype was developed by harnessing polymer configuration design with low‐entropy effect, achieving molecular‐level electrolyte engineering for excellent temperature stability of LMBs (up to 150 °C). A mechanism based on robust ion‐dipole interactions within the “Li⁺‐multivalent ester carbonyl (─C═O)‐ether (─C─O─C─)‐fluorinated (─CF_3_) segment” configuration has been elucidated and confirmed through systematic experimental and theoretical investigations. This polymer framework exhibits robust compatibility and stability regarding fluorinated‐ether copolymerization and plasticization, effectively decoupling the strong Li⁺‐ether solvation cages and facilitating rapid ion transport. Notably, the temperature‐dependent enhancement of the lithium‐ion transference number (t_Li+_) provides strong evidence for the low‐entropy‐penalty effect regulation of polymer configurations. Furthermore, the robust coordination structure ensures the high‐temperature stability of the lithium‐metal anode, as evidenced by nuclear magnetic resonance (NMR) and X‐ray photoelectron spectroscopy (XPS) analysis, which reveal the effective suppression of unfavourable side reactions between ─C═O groups/C─O─C─ chains and alkali metals. Thanks to the molecular‐level regulation, the proposed UPE achieves an exceptional ionic conductivity of 3.71 mS cm^−1^, high t_Li+_ (> 0.6, increasing with temperatures), and robust electrochemical stability at ultra‐high temperatures. The synthesized UPE enables symmetric Li metal batteries to achieve remarkable cycling stability, operating for 1000 h at 120 °C without complex interfacial reactions. Furthermore, LiFePO_4_|UPE|Li cells exhibit excellent cycling performance, sustaining over 400 stable cycles at 1.0 C at 120 °C and exceeding 100 cycles at 150 °C. This prototype design provides a foundation for advancements such as anion regulation, copolymer optimization, and the incorporation of functional additives, paving the way for innovative strategies in high‐performance, temperature‐tolerant Li metal batteries.

## 2 Results and Discussion

As shown in Figure 1b, the proposed UPE was synthesized via simple copolymerization, incorporating abundant “ester‐ether‐fluorinated segments”. Specifically, spatially adjacent ester carbonyl or ether groups in the polymer framework have been demonstrated to promote the spontaneous formation of multivalent Li^+^‐dipole interactions with a low entropic penalty. Thus, as a proof of concept, 2,2,3,4,4,4‐hexafluorobutyl acrylate (HFBA) was employed as the primary carbonyl‐rich monomer, constituting the dominant phase. Poly(ethylene glycol) diacrylate (PEGDA), featuring both carbonyl and multivalent ether groups, was employed as the crosslinker. LiTFSI provided the sole source of Li⁺ ions to generate the copolymer. Figures S1 and S2 (Supporting Information) depict the chemical reaction process with corresponding optical images before and after the reaction. The loss of fluidity in the reaction mixture provides preliminary evidence for successful copolymerization, as increased molecular weight reduces mobility. Fourier transform infrared spectra (FTIR) confirm the successful synthesis of UPE, as illustrated in Figure S3 (Supporting Information). After polymerization, the ─C═C─ peak at 1633 cm^−1^ disappears and the carbonyl peak shifts from 1730 cm^−1^ to higher wavenumbers, confirming the successful incorporation of PEGDA as a cross‐linking segment in the polymer network. As a prototype design, this formulation is expected to engineer thermally robust polymer electrolytes, achieving structural stability and decoupling strong Li⁺‐ether solvation cages for accelerated ion transport.

The mechanism of Li⁺‐multivalent ester/ether ion‐dipole interactions, which are fundamentally noncovalent, dictates the molecular design of polymers in the UPE. Figure 1c shows that the process is thermodynamically favorable (a negative Gibbs free energy, Δ*G*< 0) because of spontaneous noncovalent association under standardized conditions. The spontaneous formation process can be assessed using Equation (1):^[^ ^27^ ^]^

(1) ΔG=ΔH−TΔS<0

where Δ*H* is the change of enthalpy, *T* is the absolute temperature, and Δ*S* is the change in entropy. Δ*G* is primarily determined by Δ*H*, *T*, and Δ*S*. Typically, the maximized enthalpy gain (Δ*H*<< 0) corresponds to stronger binding interactions, higher thermostability, and more tightly coupled noncovalent bonds.^[^ ^28^ ^]^ It facilitates the formation of robust Li^+^‐coordination structures and enhances overall structural stability. Under constant pressure conditions, the Δ*S* is given by the temperature dependence of Δ*G*, based on Equation (2):^[^ ^29^ ^]^

(2) $$\Delta S=-{\left(\frac{\partial \Delta G}{\partial T}\right)}_{p}$$

The relatively low Δ*S* observed at room temperature rapidly becomes thermodynamically dominant as temperature increases due to the substantial entropy loss (Δ*S*<< 0), until the magnitude of ∣TΔ*S*∣ exceeds that of ∣Δ*H*∣, estimating the dissociation of noncovalent bonds (Δ*G*> 0).^[^ ^23^ ^]^ Thus, polymers primarily stabilized by highly ordered noncovalent hydrogen‐bond interactions exhibit limited thermal stability (typically below 100 °C), which arises from the significant entropy loss.^[^ ^30^ ^]^ To overcome these high‐temperature challenges, employing noncovalent interactions with low entropic penalties is an effective strategy. Fortunately, the densely packed Li^+^‐carboxyl or ether groups have been demonstrated to form the desired Li^+^‐dipole interactions, avoiding the need for lithium‐reactive hydrogen bonds. The Li⁺‐ether/carbonyl multivalent interactions are inherently disordered, while their strong ion‐dipole coordination promotes the formation of robust networks with self‐healing characteristics. Therefore, under high‐temperature conditions, the resulting polymer network, characterized by a slow‐growing ∣TΔ*S*∣ and a large ∣Δ*H*∣, is expected to achieve high thermal stability.

**Figure 2:**
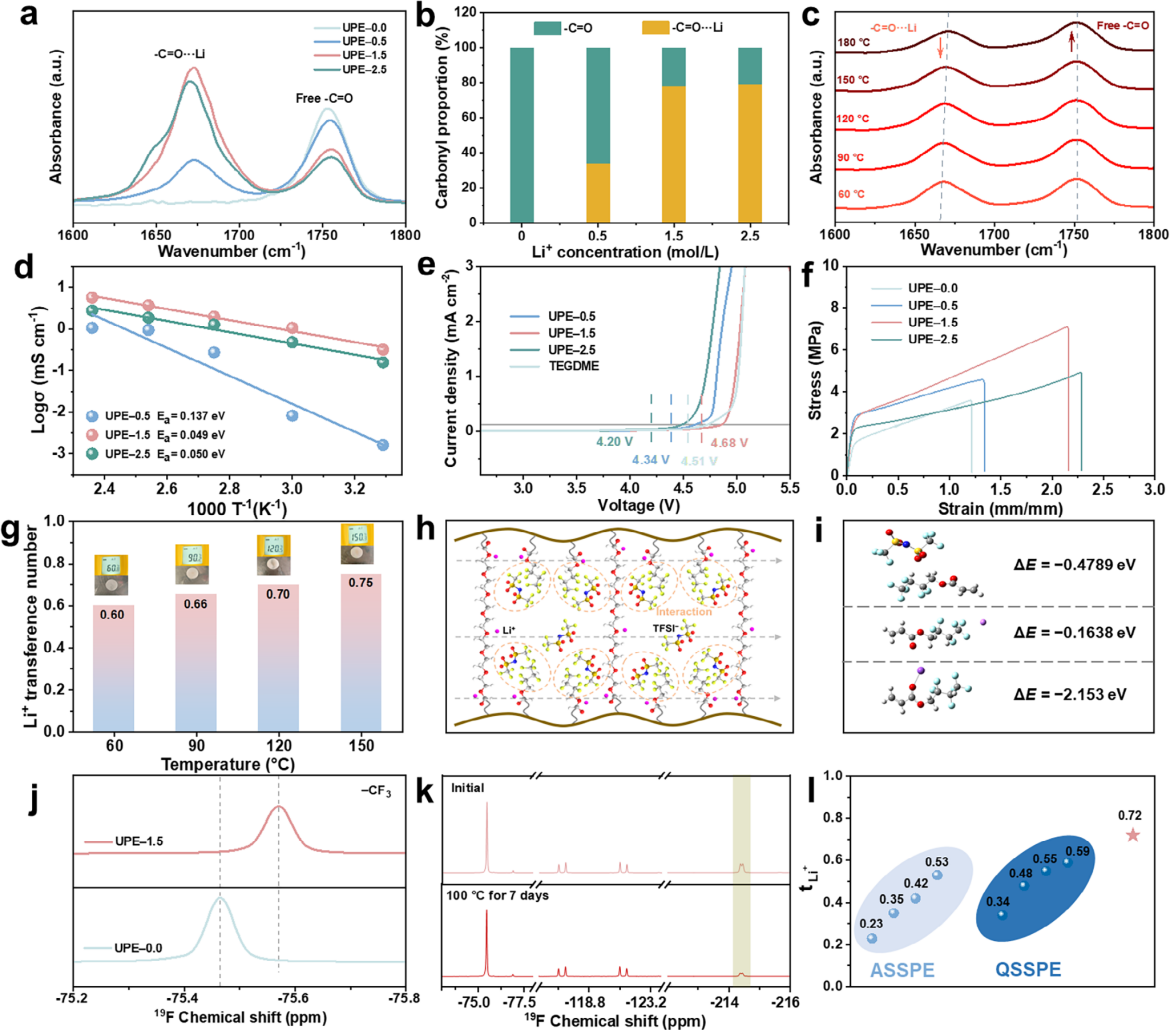
a,b) FTIR spectra and carbonyl proportion of the UPE with different LiTFSI concentrations for the C═O vibration (1600–1800 cm^−1^). c) FTIR spectra of the solvated and free carbonyl group from 60 and 180 °C. d) Arrhenius plots of the UPE with different LiTFSI concentrations, showing the ionic conductivities in the temperature range from 30 to 150 °C. e) Linear sweep voltammogram curves of the UPE with different LiTFSI concentrations and 1.5 M LiTFSI/TEGDME at 120 °C. f) Stress‐strain curves of the UPE with different LiTFSI concentrations. g) Li^+^ transfer number of the UPE‐1.5 under different high‐temperature conditions. h) Schematic diagram of the interaction between the fluorine‐containing functional groups and TFSI^−^ and Li^+^ transport pathways inside UPE. i) The binding energy between the fluorinated regions of HFBA segments with TFSI^−^ and Li⁺, and the ester functional groups of HFBA with Li⁺. j) The ^19^F NMR Spectra of UPE‐1.5 and UPE‐0.0. k) The ^19^F NMR spectra of UPE‐1.5 before and after standing at 100 °C. l) Comparison of Li^+^ transfer number of UPE, all‐solid‐state polymer electrolyte (ASSPE), and quasi‐solid‐state polymer electrolyte (QSSPE) at room temperature.

The low entropic penalty effect arising from the coexistence of multivalent Li⁺‐ether and Li⁺‐carbonyl interactions in the copolymer was confirmed by FTIR (**Figure** **2**a). The dissociation of ion‐dipole interactions leads to alterations in solvation structure, reflecting the changes in the ratio between solvated carbonyls and free carbonyls.^[^ ^31^ ^]^ The UPE‐0.0, devoid of LiTFSI, exhibits a typical stretching mode of free ester carbonyl at 1762 cm^−1^. With the addition and increase of lithium salts, a new peak emerges and intensifies at 1672 cm^−1^, which can be assigned to the solvated ─C═O structure with Li^+^.^[^ ^27^ ^]^ Deconvolution provides the area of these two peaks, enabling the calculation of the carbonyl solvation ratio (Figure 2b). These results indicate that as lithium salt concentration increases, the interactions between Li⁺ and ─C═O groups become predominant, resulting in a reduction in free carbonyl groups and enhanced electrochemical compatibility with lithium metal. It is important to emphasize that excess Li salt does not continuously augment the number or intensity of ion‐dipole interactions. Taking the lithium salt concentration at which interactions between Li⁺ and ─C═O groups reach their maximum proportion as an example (UPE‐1.5, 1.5 mol LiTFSI in the UPE), FTIR analysis further confirmed that, even at elevated temperatures, these interactions remain predominant, attesting to the persistence of a low entropy penalty in the copolymerized UPE (Figure 2c). At elevated temperatures, the solvated ─C═O exhibits higher vibrational frequencies, inducing a shift of the absorption peak toward higher wavenumbers accompanied by notable band broadening. Ultrahigh temperatures have a significant influence on the dissociation of noncovalent ion‐dipole interactions, as reflected by the decreasing relative intensity of the solvated ─C═O peak. Intriguingly, the relatively high proportion of solvated C═O remains preserved even at 180 °C. This phenomenon suggests that the ion‐dipole interactions exhibit remarkable robustness against thermal perturbations, attesting to the persistence of a low entropy penalty in the copolymer UPE electrolyte.

Component optimization was conducted using an orthogonal assay rather than an exhaustive method, enabling a comprehensive evaluation of multiple factors and levels while efficiently identifying the optimal conditions. On the one hand, the PEGDA crosslinker content is critical for achieving optimal UPE performance. Optimal loading of 5 wt.% not only promoted the formation of robust and self‐supporting films but also enhanced the successful incorporation of ether‐based plasticizers (Figures S5 and S6 and S10, Supporting Information). This synergistic effect improves ion transport, mechanical strength, and film uniformity (Figure S4, Supporting Information). On the other hand, the concentration of lithium salts presents a pronounced effect on the entropy penalty within the UPE, modulating both its mechanical and electrochemical performance. Accordingly, 0.5, 1.5, and 2.5 mol of LiTFSI were systematically incorporated into the copolymer matrix (Figures S5 and S6, Supporting Information). Interestingly, ionic conductivity does not increase monotonically with salt concentration. Incorporating 1.5 mol of LiTFSI into the UPE (namely, UPE‐1.5) results in the highest ionic conductivity at 25 °C (1.06 mS cm^−1^), 120 °C (3.71 mS cm^−1^), and 150 °C (5.76 mS cm^−1^), as shown in Figure 2d. The exceptional performance of UPE‐1.5 can be primarily ascribed to the incorporation of lithium salts, which significantly reduces the crystallinity of the copolymer membrane, promoting the formation of amorphous regions. This effect is robustly supported by both differential scanning calorimetry (DSC) and X‐ray diffraction (XRD) analyses. Specifically, DSC measurements revealed a glass transition temperature of −13.75 °C, a finding that correlates with the observed high ionic conductivity (Figure S7, Supporting Information). Moreover, XRD analysis confirmed that the presence of lithium salts diminishes crystallinity, thus facilitating enhanced ion transport (Figure S8, Supporting Information). The reduction in ionic conductivity observed for UPE‐2.5 may be attributed to an excessively high concentration of lithium salts that remain undissociated within the UPE‐2.5 matrix. This conclusion is supported by the anomalously elevated glass transition temperature of UPE‐2.5, as well as by Raman spectroscopic analysis (Figure S9, Supporting Information). Therefore, the incomplete dissociation of lithium salt at higher concentrations in UPE‐2.5 limits the availability of free Li⁺ ions for effective conduction. Consequently, UPE‐1.5 incorporating 5 wt.% PEGDA exhibits excellent ionic conductivity. Activation energy calculations from Figure 2d further indicate that the UPE‐1.5 formulation possesses a remarkably low *E_a_* value of 0.049 eV, demonstrating its outstanding performance.

Other parameters are of paramount importance to the performance of solid electrolyte membranes. Especially, the electrochemical stability window was evaluated at different temperatures (Figure 2e; Figures S10 and S11, Supporting Information). Significantly, the UPE‐1.5 exhibited a high voltage window of 4.68 V at 150 °C, markedly outperforming tetraethylene glycol dimethyl ether (TEGDME). It is worth mentioning that the UPE‐2.5 formulation exhibited a compromised electrochemical stability window, presumably due to the decomposition of the excess lithium salt. Furthermore, the mechanical properties were also evaluated. As shown in Figure 2f and Figure S12 (Supporting Information), the UPE‐1.5 formulation also demonstrated exceptional mechanical performance, with a tensile strength of 7.12 MPa and a toughness of 10.4 MJ m^−3^, confirming that a moderate Li‐salt content enhances mechanical strength. Atomic Force Microscopy (AFM) measurements further revealed that the homogeneous UPE‐1.5 membrane exhibits a Young's modulus of 6.81 MPa and an average roughness of 4.74 nm (Figure S15, Supporting Information). In addition, scanning electron microscopy (SEM) analysis corroborated that the formed membrane is dense and homogeneous (Figure S16, Supporting Information). More detailed evidence on the optimization of components was summarized in Figures S4–S18 and Table S1 (Supporting Information).

Combined with the detrimental impact of high salt concentration on maintaining a low entropy penalty, these observations suggest that high salt levels disrupt the established dynamic network, thereby increasing internal resistance and narrowing the voltage window, which results in the concurrent degradation of both mechanical and electrochemical performance. Therefore, the UPE‐1.5 exhibits a stable coordination structure even at high temperatures. A solid‐state ^7^Li NMR spectrum was employed to probe lithium‐ion migration. Figure S19 (Supporting Information) shows that UPE‐1.5 exhibits an upfield shift relative to UPE‐0.5, indicating a more mobile Li⁺ environment and complete lithium salt dissociation in the UPE‐1.5. Therefore, a unique supramolecular network was established in UPE‐1.5 through multivalent ion‐dipole interactions and crosslinking polymerization. This network endows the UPE electrolyte with exceptional ionic conductivity and a broad electrochemical stability window at elevated temperatures while maintaining mechanical robustness and self‐healing properties (Figure S20, Supporting Information).

Moreover, electrochemical impedance spectroscopy (EIS) with direct current (DC) polarization was utilized to measure the t_Li+_ of symmetric lithium cells across various high‐temperature scenarios (Figure 2g; Figure S21, Supporting Information). It is noteworthy that the t_Li+_ values for the UPE remain consistently high across all temperature conditions and further increase with rising temperature (from 60 to 150 °C), which may be attributed to the robust structure of UPE. At elevated temperatures, the polymer chains become thermally activated and softened, amplifying the effect of the plasticizer and enhancing interactions between the fluorinated side chains and TFSI^−^ anions.^[^ ^32^ ^]^ The elevated temperatures also accelerate the dissociation of Li^+^‐dipole interactions. Li^+^‐ether coordination has been shown to dissociate preferentially over Li^+^‐carboxyl coordination at elevated temperatures, resulting in the homogeneous disruption of the Li^+^‐ether cages within the robust UPE framework. Additionally, the abundant fluorinated side chains are expected to further stabilize the dissociation of these strong Li^+^‐ether coordination cages. Therefore, we can propose the interaction mechanism between the polymer chains and ions within the UPE, as shown in Figure 2h. This noncovalent polymer‐Li^+^ coordination enables a thermodynamically favorable self‐ordering during copolymerization.

Density functional theory (DFT) calculations were employed to investigate the interactions among the electrolyte components. Figure 2i illustrates that the binding energy between HFBA segments and TFSI^−^ is ≈−0.4789 eV, while the interaction between the fluorinated regions of HFBA segments and Li⁺ is comparatively weaker at −0.1638 eV. In contrast, the ester functional groups exhibit a much stronger interaction with Li⁺, reaching −2.153 eV. These findings confirm that the polymer matrix in the UPE efficiently dissociates lithium salts, with TFSI^−^ immobilized by the fluorinated side chains and Li⁺ selectively transported through coordination with ester groups. Given the inert nature of the fluorinated segments in ion transport, Li⁺ migrates independently with efficient conduction. Efficient conduction mitigates concentration polarization and interfacial side reactions in the UPE at elevated temperatures, achieving fast Li^+^ transport and cycling life. Simultaneously, solid‐state NMR (ss‐NMR) techniques were employed to confirm the results. Analysis of the ^19^F NMR peak associated with TFSI^−^ revealed an upfield shift from −79.22 ppm in pure LiTFSI to −80.38 ppm in the UPE (Figure S22, Supporting Information), confirming the presence of interactions between TFSI^−^ and the other components in the UPE. Subsequently, the ^19^F signals from the hexafluorobutyl side chains, including ─CHF─, ─CF_2_‐, and the ─CF_3_ moiety (originating from TFSI^−^ and the polymer) also exhibited upfield shifts (Figure 2j; Figure S23, Supporting Information). This collective shift indicates an increase in electron density around the fluorine atoms in TFSI^−^, confirming the strong interaction between TFSI^−^ and the fluorinated side chains of UPE. Furthermore, after storage at 100 °C for 7 days, the sample was analyzed by ^19^F NMR to evaluate the stability of this interaction. As shown in Figure 2k, the ^19^F peak positions remained stable, confirming the high‐temperature stability of the UPE coordination structure. To our surprise, a substantial decrease in the intensity of the ─CHF─ peak was observed. Given the high stability and strong polar nature of the ─CHF─ bond, this reduction may result from strong interactions between the ─CHF─ moiety and adjacent ether segments. Concomitantly, an increase in the intensity of the ─CF_3_ signal was observed, which is conducive to LiF formation in the SEI. Therefore, the robust interaction between UPE and TFSI^−^ anions promotes an elevated high t_Li+_, surpassing that of both all‐solid‐state polymer electrolytes and quasi‐solid‐state polymer electrolytes reported in previous literature at room temperature (Figure 2l; Figure S24 and Table S2, Supporting Information).

**Figure 3:**
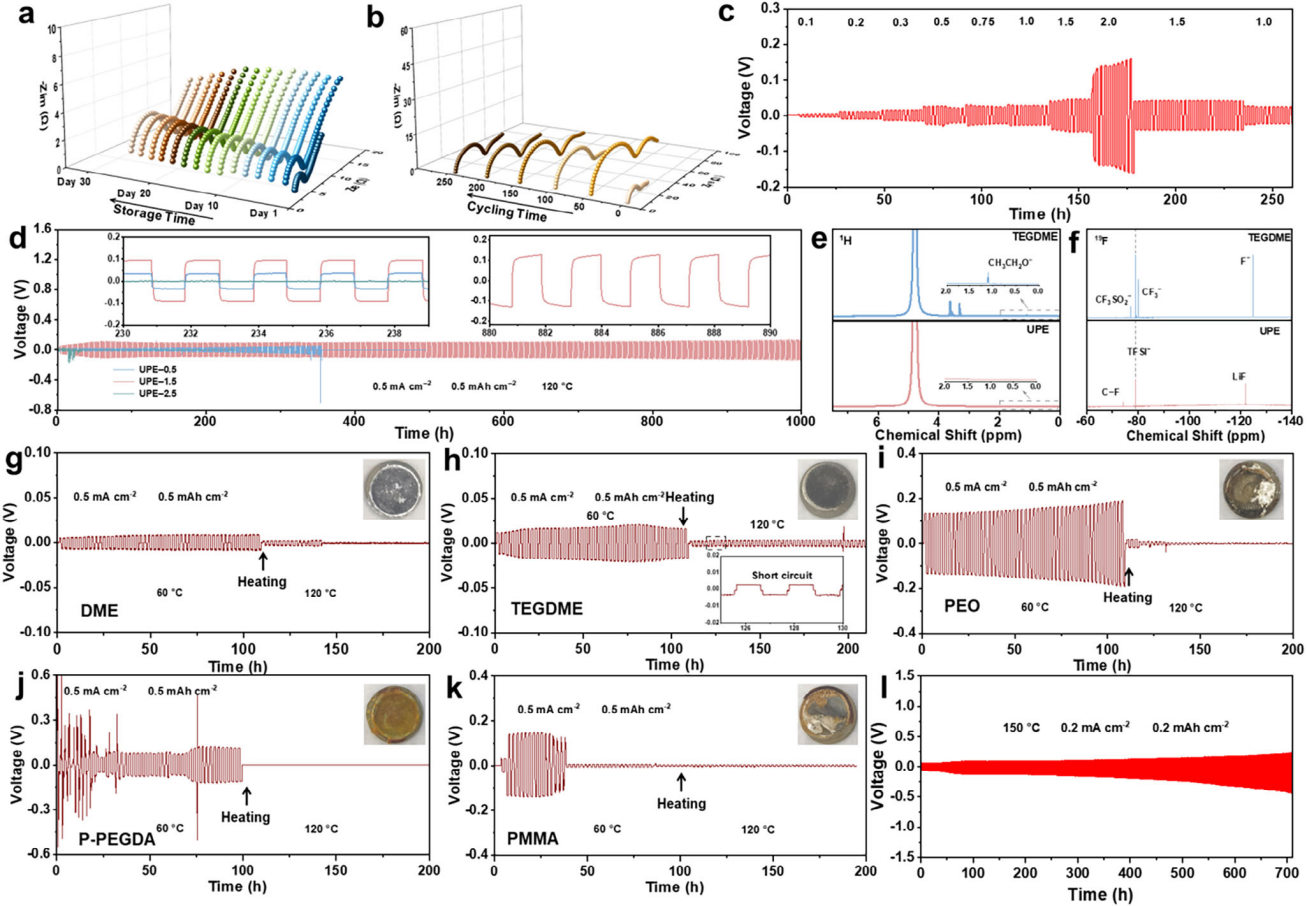
a) Impedance evolution of Li|UPE|Li battery at 150 °C during different storage times. b) Impedance evolution of Li|UPE|Li battery at 150 °C during different cycling times. c) Rate profiles of the Li|UPE|Li symmetrical battery with current densities of 0.1, 0.2, 0.3, 0.5, 0.75, 1, 1.5, and 2 mA cm^−2^ at 120 °C. d) Long‐term lithium plating/stripping experiment for symmetric Li cells with the UPE with different LiTFSI concentrations at 0.5 mA cm^−2^ and 0.5 mAh cm^−2^ at 120 °C. e, f) The surface layer of the cycled Li metal disk was scratched and dissolved into D_2_O for the NMR tests. The results are shown in the e) ^1^H NMR and f) ^19^F NMR spectra. g‐k) Galvanostatic cycling performance of the Li||Li symmetrical cells with different electrolytes including g) 1.5 M LiTFSI in DME, h) 1.5 M LiTFSI in TEGDME, i) PEO/LiTFSI, j) P‐PEGDA/LiTFSI, and k) PMMA/LiTFSI at 0.5 mA cm^−2^ and 0.5 mAh cm^−2^ at 60 and 120 °C. l) Galvanostatic cycling performance of the Li||Li symmetrical cells with UPE‐1.5 at 0.2 mA cm^−2^ with 0.2 mAh cm^−2^ capacity at 150 °C.

High temperatures present significant challenges to the stability of both the bulk phase and interfacial regions of electrolytes, particularly in the presence of high‐energy‐density lithium metal. EIS measurements serve as a crucial technique for rapidly evaluating the physical and chemical stability of the UPE under high‐temperature conditions. To assess the ion transport stability of UPE and the interfacial stability between Li metal and UPE, Nyquist plots of Li|UPE|Li symmetrical cells at 150 °C were compared over different storage durations. As shown in **Figure** **3**a, the UPE demonstrated exceptional compatibility with lithium metal anodes even under ultrahigh‐temperature conditions. The initial interfacial impedance progressively increased with storage time, rising from 2.28 to 7.27 Ω within one month. This increase is primarily attributed to the continuous and rapid formation of a stable interfacial layer resulting from the reaction between fluorine‐containing components in the UPE and Li metal surface. Additionally, the high‐frequency semicircle slowly increased over the storage period.^[^ ^33^ ^]^ Additional investigations are required to elucidate the impact of Li⁺ transport on ion‐dipole interactions and Li⁺ plating/stripping behavior within the interfacial layer during cycling. As shown in Figure 3b, the impedance of the symmetric Li battery initially increases rapidly but gradually decreases in later cycles, indicating the rapid formation of a robust and stable interfacial layer at high temperatures at 0.5 mA cm^−2^. In addition to impedance, the stability of other properties of UPE during ultrahigh‐temperature storage was further examined and presented in Figures S25–S29 (Supporting Information). The low‐entropy‐penalty in the copolymer matrix with rationally optimized component ratios exhibits exceptional operational stability under ultra‐high temperature conditions, demonstrating robust electrochemical and mechanical performance, and undamaged microstructural surface morphology through the maximized non‐covalent interactions.

Symmetric Li batteries were assembled to evaluate the interfacial stability between the UPE and Li anode, as well as their mechanical stability under ultrahigh‐temperature conditions. The critical current density (CCD) test and rate performance at 120 °C were conducted, as shown in Figure 3c and Figure S30 (Supporting Information). The corresponding current densities of 0.1, 0.2, 0.3, 0.5, 0.75, 1, and 1.5 mA cm^−2^ correspond to polarization voltages of 5.5, 11, 16, 26, 29, 32, and 46 mV, respectively. Although the overpotential increases upon reaching a current density of 2.0 mA cm^−2^, the battery remains operational, suggesting its robust tolerance under high current densities. Significantly, upon reducing the current densities to 1.5 and 1.0 mA cm^−2^, the overpotentials revert to their initial values at those conditions, indicating that neither the bulk nor the interface of the UPE undergoes severe degradation under elevated current densities. Further long‐term cycling of Li||Li cells was evaluated to assess the durability and stability of the UPE at 120 °C at 0.2 and 0.5 mA cm^−2^. (Figure 3d; Figure S31, Supporting Information). The Li||Li symmetric cell employing UPE‐1.5 maintained stable cycling for over 1000 h, exhibiting minimal polarization at 0.5 mA cm^−2^ and 0.5 mAh cm^−2^. Initially, the overpotential gradually rose from 30 to ≈100 mV, predominantly attributable to the thermal expansion of residual air in the coin cell and the progressive establishment of an interfacial layer. Charge‐discharge profiles further confirmed the stability of this SEI layer on the Li metal surface, with no indication of complex side reactions or lithium dendrite growth, which are typically observed at ultrahigh temperatures. Concurrently, at lower temperatures (25 and 100 °C), the Li||Li symmetric cell exhibited consistently low overpotentials and an extended cycling lifespan, demonstrating the superior ionic transport kinetics and robust interfacial stability of the UPE across a broad temperature range (Figures S32 and S33, Supporting Information). In contrast, the UPE‐0.5 and UPE‐2.5 counterparts were short‐circuited after 12 and 353 h, respectively, resulting in premature cell failure (Figure 3d). Figure S34 (Supporting Information) provides a comparative assessment of the lithium metal surface morphology after electrochemical cycling. The Li plating/deposition derived from the UPE‐1.5 system exhibits a distinctly compact morphology. In stark contrast, systems with low Li‐salt concentrations undergo localized electrochemical deposition at the interface, while excess Li‐salt concentrations lead to pulverization of the Li‐deposited layer.

It is essential to note that ether‐based electrolytes can dissolve lithium metal, whereas ester‐based electrolytes undergo direct reactions with lithium metal, both of which can lead to battery failure. This suggests that the low‐entropy penalty, which relies on Li⁺‐multidentate ether and Li⁺‐multidentate carboxyl coordination, may trigger deleterious reactions with lithium metal, potentially exacerbated under high‐temperature conditions. Interestingly, the remarkable compatibility of UPE with lithium metal raises our curiosity and merits further investigation. Given the high reactivity of ester groups with lithium metal, we employed ether‐based TEGDME as a comparative sample to investigate potential side reactions on the Li metal surface. Symmetric Li batteries using UPE and TEGDME were cycled at 0.5 mA cm^−2^ and 0.5 mAh cm^−2^ for 50 cycles. Following cycling, the surface layer of the Li metal was scraped off and dissolved in D_2_O for NMR analysis to identify potential byproducts. As shown in Figure 3e, the presence of characteristic byproducts CH_3_CH_2_O^−^ (1.1 ppm) on the Li metal surface matched with TEGDME confirms the formation of reactive organolithium species.^[^ ^19^ ^]^ These fragments likely originate from the C─O bond cleavage and decomposition of TEGDME during cycling, challenging the previously assumed intrinsic “inertness” of TEGDME.^[^ ^34^ ^]^ Additionally, in ^19^F NMR analysis, beyond the characteristic TFSI^−^ peak at −79.2 ppm, the control sample exhibited additional peaks at −77.1 ppm (CF_3_SO_2_^−^) and −80.1 ppm (CF_3_^−^), indicating the decomposition of TFSI^−^ on the Li metal surface (Figure 3f). Furthermore, in the TEGDME electrolyte, TFSI^−^ fails to effectively facilitate LiF formation on the Li metal surface, indicating that the inorganic LiF and organic C─F components within the SEI layer of UPE originate from HFBA rather than TFSI^−^. Ultimately, in the liquid ether‐based electrolyte, high‐temperature conditions induce the decomposition of both ether and LiTFSI, leading to the formation of reactive organolithium species and the continuous dissolution of active lithium. Unfortunately, this reaction fails to generate insoluble inorganic SEI components, leaving the process unrestrained and resulting in rapid battery failure. In contrast, UPE preferentially decomposes over other components due to its abundant fluorinated side chains, facilitating the formation of inorganic LiF, which contributes to a high‐modulus SEI and effectively prevents further undesirable reactions, thereby enhancing battery stability.

Meanwhile, liquid electrolytes and solid polymer electrolytes containing C─O and C═O bonds, as widely reported in the literature, were adopted to assemble symmetric Li||Li batteries for galvanostatic cycling tests. These tests rapidly evaluated both cycling performance and interfacial stability (Figure 3g–k; Figure S35, Supporting Information). 1,2‐Dimethoxyethane (DME) and TEGDME, benefiting from their liquid properties, exhibit lower overpotentials compared to solid PEO electrolyte at 60 °C. However, all electrolytes containing EO bonds undergo rapid short‐circuiting and fail to operate when the temperature increases to 120 °C. Similarly, solid polyester‐based electrolytes, which typically contain numerous ester groups, are unsuitable for high‐temperature applications. Therefore, solid poly(ethylene glycol) diacrylate (P‐PEGDA) and polymethyl methacrylate (PMMA) exhibited instability in cycling performance at 60 °C, with short‐circuiting occurring rapidly and leading to battery failure. In comparison, the Li|UPE|Li battery not only operated stably at 120 °C but also exhibited sustained cycling for over 700 h at 150 °C with a current density of 0.2 mA cm^−2^ and a capacity of 0.2 mAh cm^−2^ (Figure 3l).

**Figure 4:**
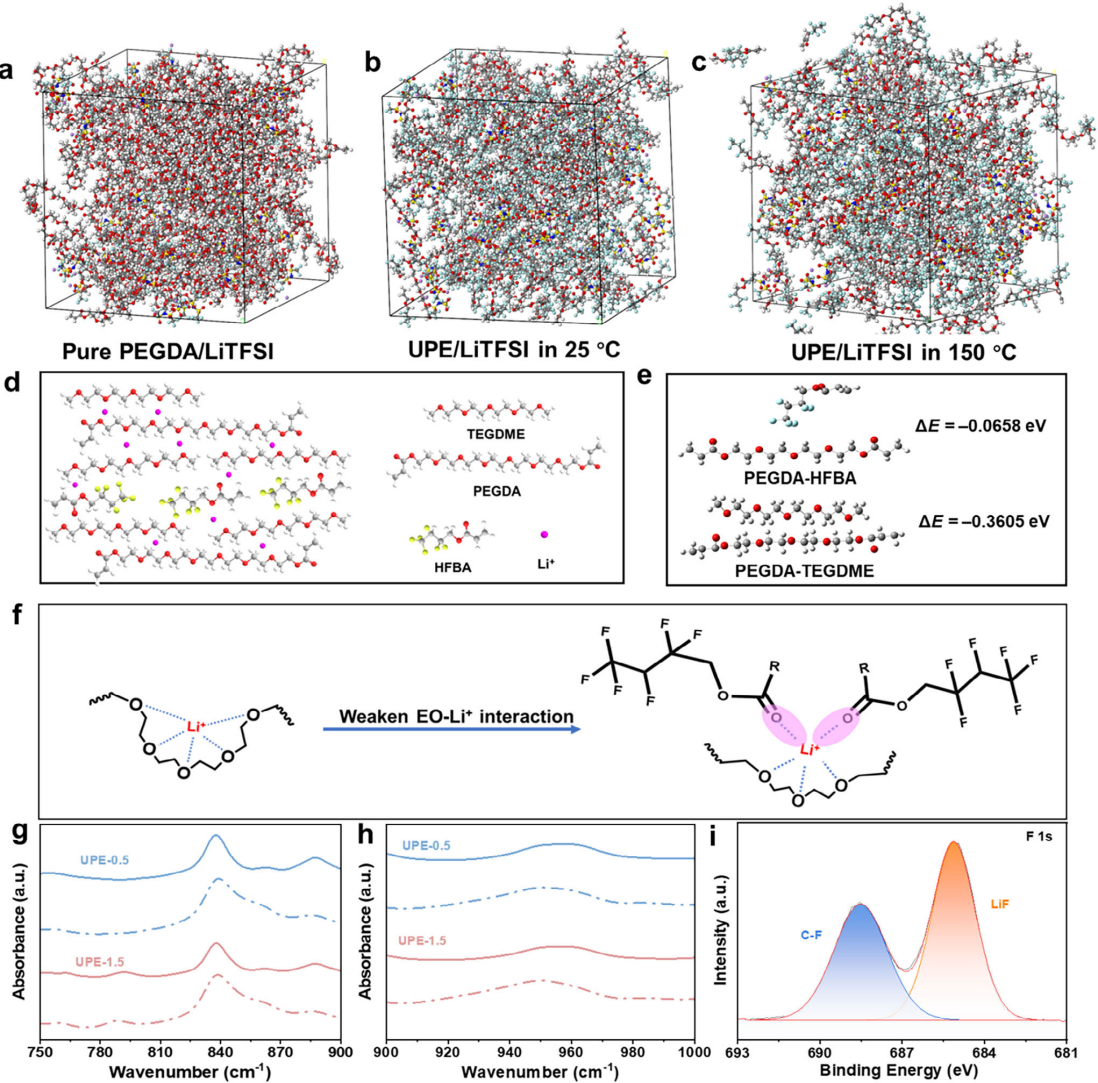
a) The simulation box of the simulation system for a) PEGDA at 25 °C, and UPE at b) 25 °C and c) 150 °C. d) Schematic diagram of the internal molecule distribution inside UPE with loosened chain entanglement. e) The binding energy of PEGDA with HFBA and TEGDME. f) Scheme of the intimate tight EO–Li^+^ interaction in UPE loosened by TEGDME. g‐h) The FTIR spectra of UPE with TEGDME (dashed line) and UPE without TEGDME (solid line) in the spectral region from g) 750 to 900 cm^−1^ and h) 900 to 1000 cm^−1^.i) F1s XPS spectra of the SEI compositions of a cycled symmetric Li cell with UPE.

The outstanding ultrahigh‐temperature stability of UPE toward lithium metal indicates that its copolymeric architecture and Li^+^ coordination environment are fundamentally distinct from those of its individual constituent components. Molecular dynamics (MD) simulations were performed to investigate the low‐entropy penalty mechanism governing polymer chain arrangement under ultrahigh‐temperature conditions.^[^ ^35^ ^]^ As shown in **Figures** **4**a and S36 (Supporting Information), the molecular distribution in pure PEGDA (containing carbonyl and ether bonds) and LiTFSI reveals that EO chains are highly entangled and tightly coordinated with Li⁺. This highly disordered system negatively impacts both mechanical stability and ionic transport. Figure 4b illustrates that within the UPE system, all molecules and EO chains exhibit an independent and well‐ordered distribution. The interaction between polymer monomers and Li⁺ effectively reduces the disordered entanglement among polymer chains, promoting a more organized structure. At 150 °C, despite localized molecular disordering at the model periphery induced by thermal stress, the UPE system retains a well‐ordered distribution, attributable to persistent monomers and Li^+^ interactions under extreme conditions (Figure 4c).

TEGDME molecules are uniformly dispersed among acrylate monomers, with ether bonds facilitating the loosening of entanglements between the polymer chains, as shown in Figure 4d.^[^ ^14^, ^36^ ^]^ According to the principle of “like dissolves like,” TEGDME molecules are uniformly dispersed among PEGDA chain segments. The binding energies between PEGDA and HFBA or TEGDME further validated this proposed mechanism (Figure 4e). PEGDA exhibited a higher binding energy with TEGDME (−0.3605 eV), leading to the preferential attraction of TEGDME molecules in its vicinity. Consequently, PEGDA segments within the copolymer act as templates or storage spaces for TEGDME as a plasticizer. Due to its minimal occupancy (5 wt.%), PEGDA maintains a highly homogeneous distribution. Meanwhile, the abundant fluorinated side chains, introduced through copolymerization, effectively segregate TEGDME and PEGDA, preventing spontaneous entanglement. Additionally, the competitive interaction between ester and ether groups further disrupts the coordination environment, inhibiting the formation of strong Li⁺‐ether coordination cages within the UPE matrix (Figure 4f). This structural regulation prevents EO chains from bending and entangling within the 3D polymer network, while simultaneously shielding fragile carbonyl groups from decomposition at the interface under ultrahigh temperatures, enhancing the thermal and electrochemical stability of the system.

To verify the evolution of solvation cages in the UPE matrix, FTIR was collected to reflect molecular conformational changes. Figure 4g,h shows the spectrum ranging from 750 to 1000 cm^−1^, where the absorption peaks contained a typical ─CH_2_‐ rocking vibration (838 cm^−1^) and a stretching vibration of C─O (955 cm^−1^). These two peaks were sensitive to torsional angle conformational variations of the ─O─C─C─O─ segments. With increasing Li⁺ concentration, the peaks varied to display a sharp shape, indicating that the enhanced Li^+^ coordination caused limited conformational flexibility. Strikingly, upon TEGDME addition (dashed lines), the peak at 838 cm^−1^ broadens substantially at each Li⁺ concentration, suggesting a larger torsion angle and conformational change within the EO chains. This phenomenon directly evidences the loosened chain entanglement of TEGDME inside the UPE. Further corroboration arises from the C─O stretching vibration at 955 cm^−1^ (Figure 4h). The red shift of the peak in TEGDME‐containing UPE confirms the weakened Li^+^‐ether coordination strength, further testifying to the ability of TEGDME to decouple the Li^+^‐ether solvation cage and influence the ionic coordination environment.

To date, we have demonstrated that by harnessing the low entropy penalty effect, it is successful to simultaneously incorporate both Li⁺‐multivalent ether and Li⁺‐multivalent ether interactions within a single system. This dual functionality enables exceptional ionic transport and outstanding ultrahigh‐temperature stability in contact with lithium metal. XPS was utilized to evaluate the interfacial stability of the system with carboxyl‐rich and ether‐rich groups. The chemical composition of the SEI layer in symmetric Li cells with UPE after 50 cycles was analyzed. In the XPS C1s spectrum (Figure S37a, Supporting Information), a prominent ─C─O─ peak (≈286.4 eV) appears and primarily originates from the EO bonds of UPE, indicating the presence of a polyether‐rich layer. Figure 4i exhibits prominent C─F and LiF peaks, verifying the presence of a fluorine‐rich organic‐inorganic hybrid interfacial layer. Combined with the previous discussion of fluorine‐containing functional groups within the cross‐linked polymer network, the shift in electron distribution within the ─CHF─ moiety brings the paired electrons closer to the carbon chain rather than the F atom, thereby enhancing the susceptibility of the F atom to detachment and facilitating the LiF‐rich SEI formation under electrochemical conditions. (Figure S37b, Supporting Information).

**Figure 5:**
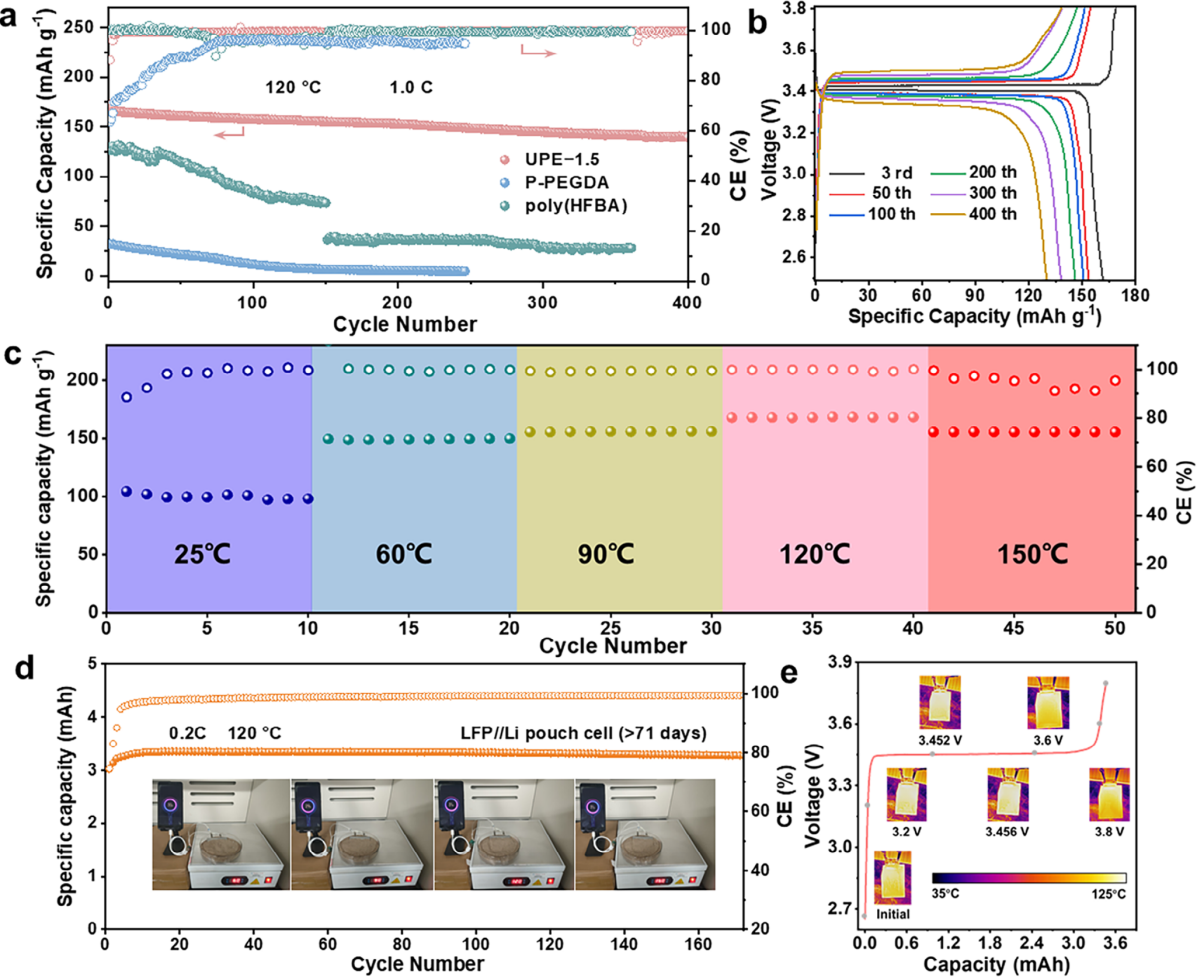
a) Cycling performance of LFP||Li with UPE‐1.5, P‐PEGDA and poly(HFBA) at 1.0 C at 120 °C. b) Charge/discharge curves of LFP|UPE‐1.5|Li cells. c) Cyclic performance of LFP|UPE‐1.5|Li under varying temperature conditions. d) Cycling performance of an LFP|UPE‐1.5|Li pouch cell at 0.2 C at 120 °C, the insets are a series of optical images of the phone charged by an LFP|UPE‐1.5|Li pouch cell at 60, 90, 120, and 150 °C. e) Infrared thermography of the pouch cell during the charging process from 2.5 to 3.8 V.

Considering the performance characteristics of ionic conductivity, along with electrochemical stability and mechanical properties, solid‐state LMBs employing LiFePO_4_ (LFP) cathodes with active material mass loadings of ≈ 3.4 mg cm^−2^ were assembled and measured to evaluate the cycling performance at ultrahigh temperature. The LFP|UPE|Li batteries were cycled between 2.5 and 3.8 V at 120 °C. The Cyclic voltammetry (CV) curves exhibited excellent reversibility and interfacial stability (Figure S38, Supporting Information). As depicted in **Figure** **5**a, under consistent experimental conditions, the long‐term cycling performance of the two contrast sample concentrations of UPE exhibited significantly inferior outcomes. Under insufficient lithium salt concentration, as exemplified by UPE‐0.5 (Figure S39, Supporting Information), the cell was nearly incapable of sustaining normal charge‐discharge cycles at 120 °C, rapidly succumbing to short‐circuiting. This failure is attributable to inadequate Li⁺‐dipole interactions, which fail to sustain an effective ion transport pathway under high‐temperature conditions. By contrast, although UPE‐2.5 sustained cycling at 120 °C, its specific capacity was significantly reduced (Figure S39, Supporting Information). This diminished performance is primarily attributable to the high salt concentration, which impedes the Li⁺‐dipole interactions essential for establishing an efficient ion transport network. Remarkably, the LFP|UPE‐1.5|Li full battery demonstrated superior cycling stability, retaining a capacity of 139.7 mAh g^−1^ after 400 cycles at 1.0 C, and exhibited excellent rating charging and discharging capabilities. In contrast, P‐PEGDA (containing abundant ester and ether groups) and poly(HFBA) (reliant on ion‐dipole interactions and fluorine‐containing functional groups) exhibited low initial capacities and rapid capacity degradation. This performance disparity originates from their structural limitations: the rigid polar networks of P‐PEGDA impede ion transport kinetics, while the inadequate mechanical robustness of poly(HFBA) compromises the long‐term cycling stability of devices under elevated temperatures. The associated charge‐discharge profiles corroborated the exceptional cycling stability (Figure 5b). Furthermore, the cell could provide excellent rate capabilities of 169.4, 167.5, 166.4, 166.1, and 165.1 mAh g^−1^ at varying current densities of 0.2, 0.5, 1.0, 1.5, and 2.0 C, respectively (Figure S40, Supporting Information). And the initial capacity of the cell can be recovered at 1.0 C, demonstrating the stable Li^+^ transport performance of the UPE at ultrahigh temperatures. The cycle performance of the UPE remained outstanding even when the cathode was loaded with a higher amount (≈9.65 mg cm^−2^) of active material at 120 °C (Figure S41, Supporting Information). Meanwhile, as shown in Figure S42 (Supporting Information), LFP|UPE‐1.5|Li full battery not only demonstrated stable electrochemical performance under ultrahigh temperature conditions but also exhibited high specific capacity and stable cycle life at lower temperatures (25, 60, and 100 °C). Even at 150 °C, the battery at 0.6 C retained a capacity of 119.8 mAh g^−1^ after 100 cycles (Figure S43, Supporting Information). In contrast, LFP||Li cells utilizing commercial liquid electrolytes and PVDF‐based SPE are unable to complete a full charge‐discharge cycle even at high temperatures (Figure S44, Supporting Information).

Figure 5c illustrates the robustness of the cell against external temperature change. Upon a gradual increase in ambient temperature from 25 to 150 °C, the LFP|UPE|Li battery maintains stable cycling and discharging performance, indicating the superior electrochemical properties of UPE across a broad temperature range. This characteristic is crucial for ensuring battery lifespan in practical applications exposed to environmental temperature variations.^[^ ^37^ ^]^ Additionally, pouch cells of LFP|UPE|Li were fabricated to further demonstrate the thermal stability of UPE. As illustrated in Figure 5d, the pouch cell assembled with UPE exhibits a notably long operational lifespan, preserving substantial capacity without significant decay over more than 170 cycles. Experimental results demonstrate that both coin cells and pouch cells exhibit significantly superior cycling stability to previously reported lithium batteries under high‐temperature conditions (Table S3, Supporting Information).^[^ ^38^, ^39^, ^40^, ^41^, ^42^, ^43^, ^44^, ^45^, ^46^, ^47^, ^48^, ^49^ ^]^ Furthermore, the assembled pouch cell can consistently deliver power over a wide range of high temperatures from 60 to 150 °C, enabling sustained energy provision for mobile phones. Ultrahigh operating temperatures pose significant challenges to battery thermal runaway mechanisms. Consequently, we employed real‐time temperature observation of a pouch cell during the charging process at various voltage points using a thermal infrared camera (Figure 5e). The high thermal resistance pouch cell exhibits minimal temperature changes and the absence of hotspots during the charging process, suggesting that the wider electrochemical stability window of the UPE can reduce the thermal runaway risk.

## 3 Conclusion

In conclusion, we have developed a robust UPE that enables stable, long‐term cycling performance of solid‐state lithium metal batteries under extreme thermal conditions. The UPE was synthesized through the copolymerization of “ester‐ether‐fluorinated segments,” leveraging the spatial proximity of carboxyl and ether groups to establish Li⁺‐multivalent carboxylate and Li⁺‐multivalent ether ion‐dipole interactions with minimal entropic penalty. This molecular‐level design endows the UPE with exceptional thermal stability (up to 150 °C), high ionic conductivity (1.06 mS cm^−1^ at 25 °C; 5.76 mS cm^−1^ at 150 °C), and an elevated Li⁺ transference number (0.60–0.75, increasing with temperature). Experimental and computational studies confirm that the low‐entropy penalty in the UPE, enabled by the “Li⁺‐multivalent carboxylate” and “Li⁺‐multivalent ether” copolymer architecture, maintains robust Li⁺ ion‐dipole interactions under extreme thermal conditions, ensuring superior mechanical and electrochemical performance. Furthermore, the incorporation of fluorine‐rich side chains reduces Li⁺‐ether entanglements, facilitating efficient Li⁺ transport. As a result, LiFePO_4_|UPE|Li full cells demonstrated outstanding cycling performance, maintaining over 400 stable cycles at 1 C and 120 °C, and surpassing 100 cycles at 150 °C. Notably, the LiFePO_4_ pouch cell also demonstrates stable operation at 120 °C. This design prototype effectively addresses the longstanding challenge of polymer‐based electrolyte degradation under elevated and ultrahigh temperatures, offering versatility through the flexible substitution of salts and plasticizers. Our molecular‐level polymer configuration design represents a robust and scalable strategy for developing high‐performance polymer‐based lithium metal batteries.

## Conflict of Interest

The authors declare no conflict of interest.

## Supporting information



Supporting Information

## Data Availability

The data that support the findings of this study are available in the supplementary material of this article.
