# Supplementary material for: Ultrahigh‐Temperature‐Tolerance Lithium Metal Batteries Enabled by Molecular‐Level Polymer Configuration Design with Low‐Entropy‐Penalty Effect
Source: Adv Sci (Weinh). 2025 Aug 16;12(41):e07191. doi: 10.1002/advs.202507191 (PMC12591149; doi:10.1002/advs.202507191)
Supplement: Supplementary file 1 — Supporting Information [file ADVS-12-e07191-s001.docx]

Supporting Information

Ultrahigh-Temperature-Tolerance Lithium Metal Batteries Enabled by Molecular-Level Polymer Configuration Design with Low-Entropy-Penalty Effect

Weiting Ma ^a^, Shunshun Zhao ^a^, Shuang Wan ^a^, Jiajun Gong ^a^, Sinian Yang ^a^, Yong Chen*^, b^, Shimou Chen*^, a^, and Guoxiu Wang*^, b^

[a] Weiting Ma, Shunshun Zhao, Shuang Wan, Jiajun Gong, Sinian Yang, and Shimou Chen

State Key Laboratory of Chemical Resource Engineering, Beijing Key Laboratory of Electrochemical Process and Technology of Materials

Beijing University of Chemical Technology,

Beijing 100029, P.R. China.
E-mail: [chensm@buct.edu.cn](mailto:chensm@buct.edu.cn)

[b] Yong Chen, Guoxiu Wang

Centre for Clean Energy Technology

University of Technology Sydney

Broadway, Sydney, NSW 2007, Australia

E-mail: Yong.chen@student.uts.edu.au; [Guoxiu.Wang@uts.edu.au](mailto:Guoxiu.Wang@uts.edu.au)

# Experimental Procedures

**Materials**

The following chemicals were directly utilized without further purification upon receipt: 2,2,3,4,4,4-hexafluorobutyl acrylate (HFBA, 98%, Bidepharm), poly(ethylene glycol) diacrylate (PEGDA, Mw = ~400, Aladdin), 2-Hydroxy-2-methylpropiophenone (HMPP, ≥97%, Aladdin), tetraethylene glycol dimethyl ether (TEGDME, ≥ 99.5%, Aladdin), polyacrylonitrile (PAN, Mw = ~200000, Sigma Aldrich), N, N-Dimethylformamide (DMF, 99.8%, Aladdin), N-methylpyrrolidone (NMP, 99.9%, Aladdin), lithium bis(trifluoromethanesulphonyl)imide (LiTFSI, 99.9%, Solvay), poly(vinylidene fluoride) (PVDF, Macklin).

**Preparation of the PAN fiber membrane**

Before use, the PAN powder should be vacuum-dried at 60°C for 24 h. Then PAN solution with a mass fraction of 12 wt.% was prepared by dissolving the 0.5 g PAN polymer powder in the DMF solvent, followed by mechanical stirring for 4 h at 60°C and ultrasonic stirring for 1.5 h. The electrospinning of PAN precursor solution was carried out at a supply speed of 1.1 mL/h and applied voltage in the range of 13.5 kV. The spinneret tip-to-collector distance was 15 cm, and the Al foil was used as the collector for PAN nanofibers. The operating temperature was maintained at ≈26±3 ℃ and the humidity during the preparation was kept at ≈23 ± 5%. Then, the electrostatic spinning PAN membrane was dried under vacuum at 80 ℃ for more than 24h to remove the residual solvent.

**Preparation of the UPE**

0.03 g HMPP, 0.5 g TEGDME, and 5 wt.% PEGDA were dissolved in 2 mL HFBA and stirred at room temperature for 4 h until the solution was completely transparent. 0.861 g LiTFSI (1.5 mol) was added to the mixed solution and stirred for 1h until transparent. After evenly laying the PAN fiber membrane onto the PTFE mold, the precursor solution was cast on the PAN surface of a well-polished Teflon plate. The polymerization was light-initiated under UV light irradiation (wavelength 365 nm) for 30 min to obtain the cast film. The thickness of the prepared UPE film was controlled at ~ 150 μm and was cut into 16mm discs for use. The preparation process for the P-PEGDA control sample involved substituting the HFBA monomers with an equivalent amount of PEGDA, while keeping all other preparation steps unchanged. For the Poly(HFBA) control sample, the preparation process excluded the addition of PEGDA, with all other preparation steps unchanged. The stirring and polymerization processes were carried out in a glove box (Mikrouna, [O_2_] < 0.1 ppm, [H_2_O] < 0.1 ppm) filled with ultrapure argon gas (≥99.999%), with close attention to interference from other volatile reagents. To further illustrate the advantage of UPE, we prepared different control samples of solid-state electrolytes and liquid electrolytes separately. Among them, the concentration of LiTFSI in the liquid electrolyte was uniformly maintained at 1.5 mol L^−1^. The preparation process of the PEO/LiTFSI electrolyte involves dissolving PEO and LiTFSI in acetonitrile at a ratio of EO: Li = 15: 1, and then directly scraping and coating it on a glass plate or casting it on the PAN fiber substrate to obtain an electrolyte film. The preparation process of the PMMA/LiTFSI electrolyte involves dissolving PMMA and LiTFSI in DMF at a mass ratio of 7: 3 and then casting it onto the PAN fiber substrate to obtain an electrolyte film. The preparation process of the PVDF electrolyte involves dissolving PVDF and LiTFSI in NMP at a mass ratio of 7: 3 and then casting it onto the PAN fiber substrate to obtain an electrolyte film. The thickness of the above-mentioned solid-state electrolyte films was controlled to be ~150 μm, and were cut into 16 mm discs for use. The commercial liquid electrolyte mentioned in the text is 1.0 mol L^−1^ LiPF₆ in EC/DMC/EMC (1:1:1).

**Materials Characterization**

X-ray diffractometry (XRD) was carried out by the X-ray powder diffractometer (D8 ADVANCE, Bruker AXS GmbH Co., Ltd) equipped with a Cu Kα radiation (λ = 0.15406 nm) source (40 kV, 200 mA). The morphology and structure of the UPE were characterized by a Scanning Electron Microscope (SEM, Hitachi SU8020 Japan) with an acceleration voltage of 15 kV and an atomic force microscopy-infrared spectroscopy (AFM-IR, Bruker, Anasys nanoIR3). Fourier transform infrared (FTIR) spectra were recorded with a Thermo Fisher Nicolet Antaris II infrared spectrum analyzer with the wavenumber from 4000 to 500 cm^−1^. Gaussian-Lorentzian fitting was used to calculate the areas of the FT-IR peaks. Solid-state NMR analysis was performed on a Bruker AV II-600 MHz equipped with a 5 mm probe in a static state. Before conducting solid-state NMR testing, UPE film samples need to be crushed and ground. The specific method involves using scissors to break the film into small pieces, which are then placed in liquid nitrogen for freezing before being ground. This process is repeated several times to obtain a powdered sample. To investigate the thermal stability of the UPE, thermogravimetric analysis (TGA) measurement was carried out on a TA600 instrument under N_2_ atmosphere from 50~800 ℃ at 20°C min^−1^. Differential scanning calorimetry (DSC) was tested using a Q1000 Modulated Differential Scanning Calorimeter (TA Instruments) under flowing N_2_ at 5 ℃ min^−1^. The stress-strain tests were carried out by using a dynamic mechanical analysis (DMA, WATERS Q800) tool with a tensile speed of 2 mm min^–1^. All the samples were prepared with a length of 40.0 mm and a width of 5.0 mm. Tensile tests were conducted using Sunstest UTM2502, with a constant strain rate of 100 mm min^–1^ under ambient conditions (25°C, RH 10%). The cyclic tensile stress-strain curves were obtained at the strain rate of 100 mm min^–1^

**Fabrication of Cells**

The LiFePO_4_ (LFP) cathodes were prepared by mixing LFP powder, conductive carbon black (Super P, Aladdin), and polyvinylidene difluoride (PVDF, Macklin) binder at a weight ratio of 8:1:1 in NMP solvent to form a smooth slurry. The slurry was then magnetically stirred for 12 h and cast onto carbon-coated aluminum foil. After drying at 80°C in a vacuum oven overnight and removing the NMP solvent, the LFP-based cathode layer was punched into disks (12 mm in diameter), with a common mass loading of over 3.4 mg cm^−2^ LFP foil. Coin cells (CR2025) were assembled with a Li-metal anode, LFP as the cathode. LFP||Li pouch cells (3×4 cm^2^) were prepared in an Al-laminated film battery casing. The battery fabrication processes were performed inside a glove box (Mikrouna, [O_2_] < 0.1 ppm, [H_2_O] < 0.1 ppm) filled with ultrapure Ar (≥99.999%).

**Electrochemical Characterizations**

The ionic conductivity σ of the UPE was obtained from symmetric stainless steel (SS) cells by electrochemical impedance spectroscopy (EIS) using a Metrohm Auto lab M204 electrochemical workstation (Nova 2.1) in the frequency range of 1 MHz to 0.1 Hz with an AC amplitude of 10 mV. The ionic conductivity σ was calculated using σ = *d*/*RS*, where *d* is the thickness of the UPE, *S* is the contact area between UPE and SS, and *R* is the resistance measured from EIS. The activation energy *E*_a_ of the electrolyte was obtained from the Arrhenius equation^[1]^, σ(T)=Aexp(−*E*_a_ /RT), where A is a pre-exponential factor, R is the molar gas constant (8.314 J mol^−1^ K^−1^), *Ea* is the activation energy and *T* is the absolute temperature. The Li^+^ transference number *t*_Li+_ was obtained using a symmetric Li cell by a chronoamperometry test with a DC voltage amplitude of 10 mV. The EIS before and after DC polarization was acquired in the frequency range of 0.1 Hz to 1 MHz. The value of *t*_Li+_ can be calculated according to *t*_Li+_=*I_ss_*(Δ*V*−*I_0_R_0_*)/(*I_0_* (Δ*V*−*I_ss_R_ss_*))^[2]^, where *I_0_* and *I_ss_* are the initial and steady-state currents measured by chronoamperometry, *R_0_* and *R_ss_* are the initial and steady-state resistances from EIS. The linear sweep voltammogram (LSV) analysis was conducted between 2.8 and 6.0 V at a scan rate of 1 mV s^−1^ with a Li/SS asymmetric cell to measure the electrochemical window. The cyclic voltammetry (CV) experiments of the LFP|UPE|Li cell were conducted on an electrochemical workstation from 2.5 V to 3.8 V at a scan rate of 0.1 mV s^−1^. The compatibility of UPE with a Li anode was evaluated by stripping/plating experiments with Li||Li CR2032 coin cells assembled and further evaluated using a battery testing system (CT2001A, LANHE) under different current densities and different temperatures. Galvanostatic cycling tests were further conducted at different temperatures and different rates between 2.5 to 3.8 V for LFP||Li (1 C = 170 mA g^−1^).

**Computational Methods**

Force-field molecular dynamics (FFMD) simulations were carried out using the MD software Material Studio to provide insights into the UPE designed in this work. The forced field parameters were obtained from the COMPASS III force field. Initially, 226 HFBA, 12 PEGDA, 43 TEGDME, and 57 LiTFSI molecules were packed into a 4×4×4 nm^3^ box to simulate UPE. For comparison, the pure PEGDA was simulated by packing 97 PEGDA and 57 LiTFSI molecules in a 4×4×4 nm^3^ box. The periodic boundary conditions were set in all three directions. Molecular dynamics simulations were conducted on two systems with different compositions using the Forcite module in Materials Studio. The COMPASS III force field was employed in the molecular dynamics simulations, and structural optimizations were performed for each system to obtain stable configurations with lower system energies. The electrostatic interactions were treated using the Particle–Mesh–Ewald method^[3]^. The temperature was controlled by coupling the system with a Nosé–Hoover thermostat^[4]^. The pressure was controlled using the Parrinello–Rahman pressure^[5]^. Quantum chemical calculations were performed using Gaussian 09 software. Molecular structures were visualized, built, and initially analyzed with GaussView 6.0. Subsequent geometry optimizations were conducted employing the B3LYP hybrid functional in conjunction with the 6-311G(d,p) basis set to locate the energy minima.


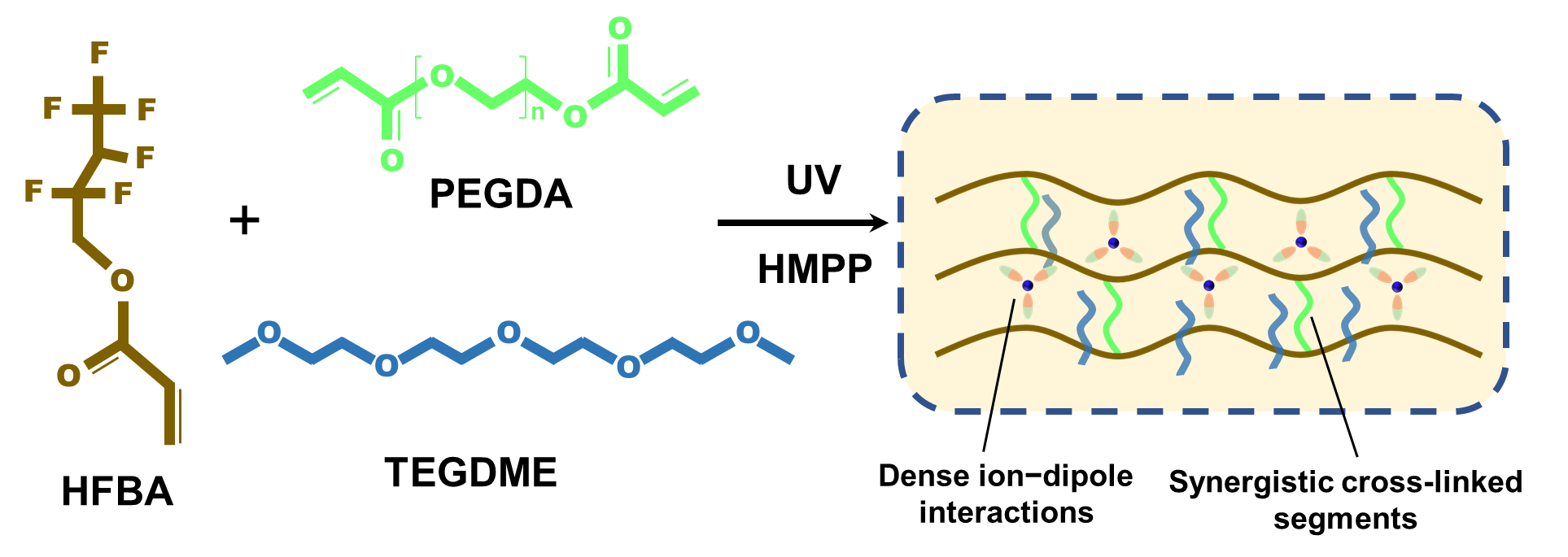


**Figure S1.** Main chemicals and synthesis scheme for the preparation process of the cross-linked UPE. Under thermal conditions, the segments situated between two adjacent cross-links are highly activated, leading to fast softening of the whole network.


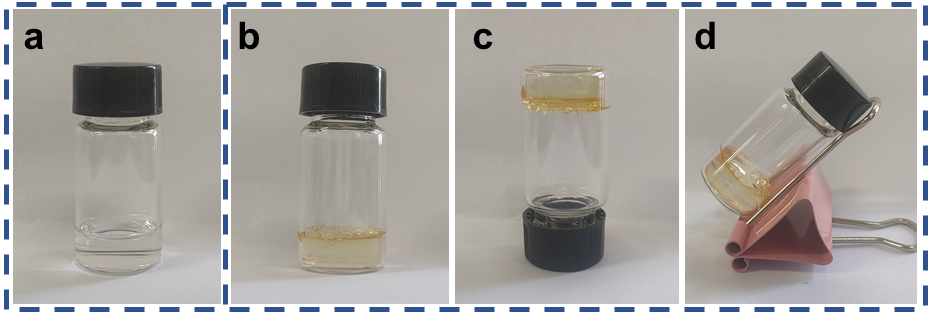


**Figure S2.** Pictures of the state of UPE precursor solution without PAN fibers a) before and b-d) after photopolymerization.

**Figure S3.** FTIR spectra of HMPP, TEGDME, PAN, PEGDA, HFBA, and UPE with a wavenumber range of 500−2100 cm^−1^.


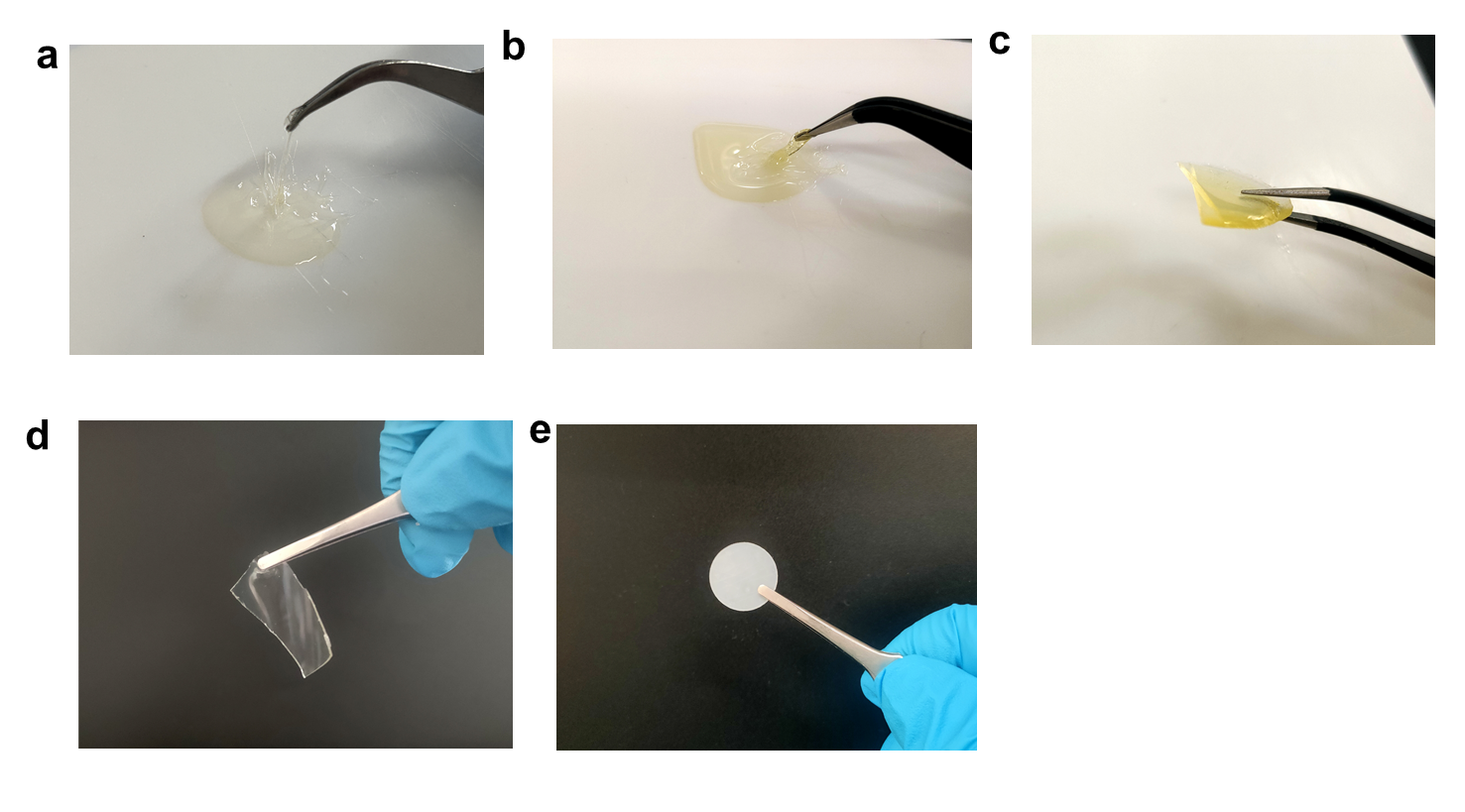


**Figure S4.** a-d) Digital images of three kinds of poly(HFBA) including a) without PEGDA, b) with TEGDME, c) 20 wt% PEGDA, and d) 5 wt% PEGDA. e) Digital images of UPE-1.5.

In the absence of multivalent ether-based crosslinkers, the copolymer exhibits a low ionic conductivity. Moreover, the electrolyte without the PEGDA crosslinker exhibits viscous polymeric characteristics, which hinders film formation (Figure S4a). Apart from the copolymer approach, Figure S4b further demonstrates that incorporating tetraethylene glycol dimethyl ether (TEGDME), a high-temperature-stable ether solvent, as a plasticizer into the pure poly(HFBA) electrolyte leads to severe phase separation, primarily due to the incompatibility between the ether moieties and the fluorinated side chains. Thus, incorporating the PEGDA crosslinker facilitates the integration of ether-based plasticizers. However, excess PEGDA would compromise the mechanical integrity of the copolymer (Figure S4c). Thus, PEGDA was incorporated at a typical crosslinker loading of 5 wt%, which facilitated the formation of self-supporting films (Figure S4d).


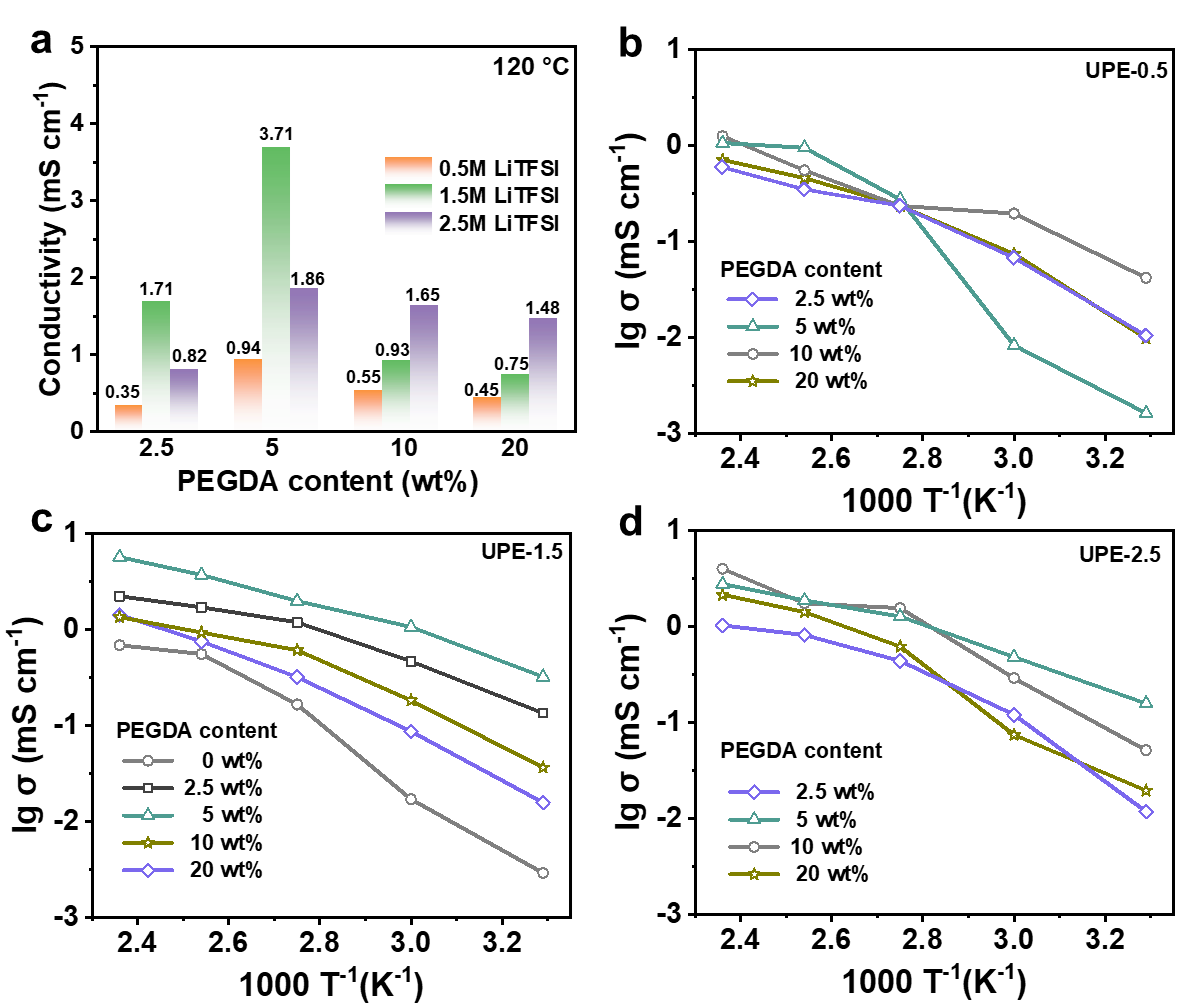


**Figure S5.** a) Comparison of the ionic conductivity of UPE-0.5, UPE-1.5, and UPE-2.5 with varying PEGDA content at 120°C and (b-d) corresponding Arrhenius plots.

**
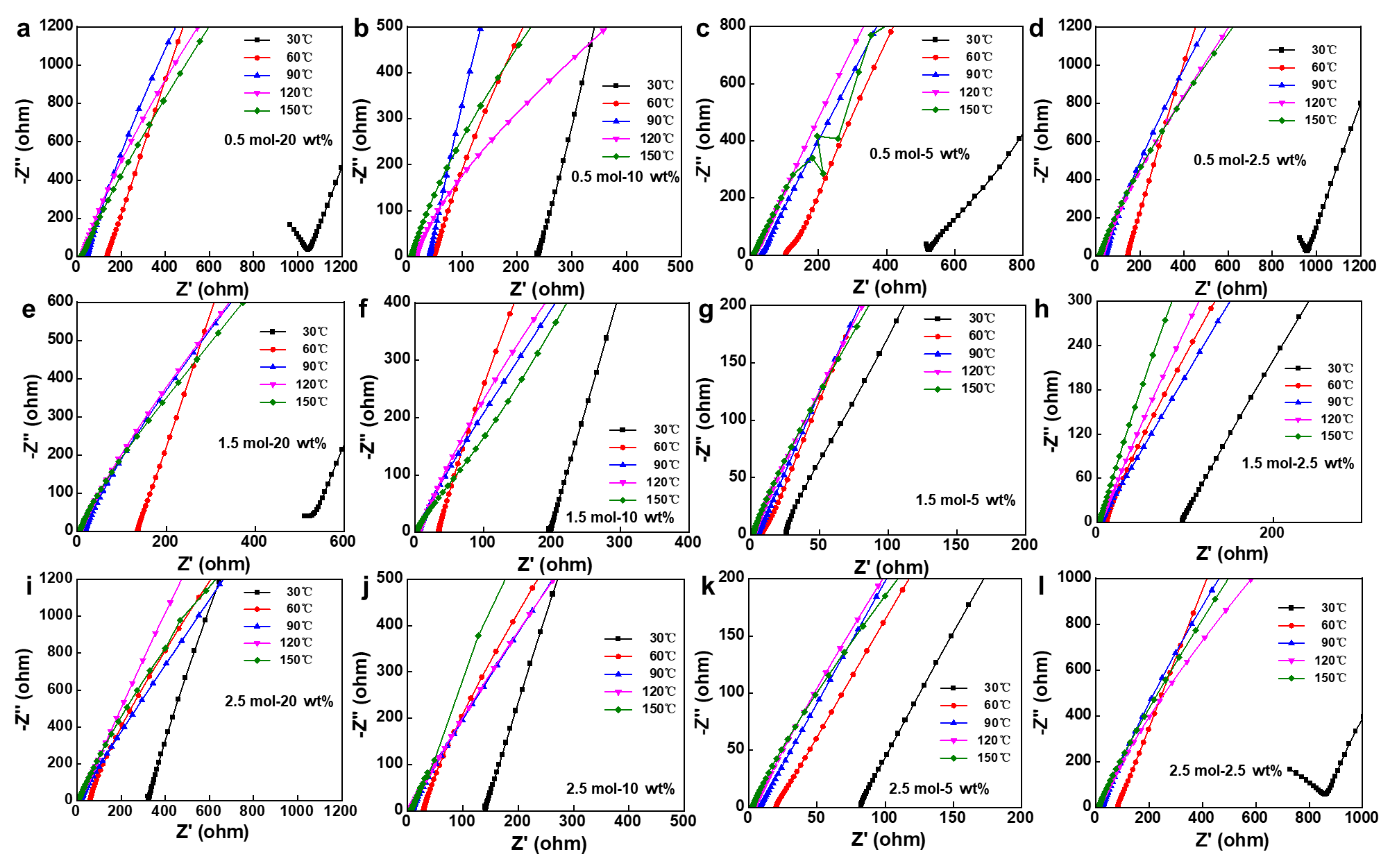
**

**Figure S6.** Impendence spectra of different UPE with varying PEGDA content and at different temperatures: (a-d) UPE-0.5, (e-h) UPE-1.5, (i-l) UPE-2.5.

**Table S1.** To obtain a reliable PEGDA additive amount, we designed experiments across multiple UPE groups with varying lithium salt concentrations to investigate the impact of varying PEGDA concentrations on the ionic conductivity of polymer electrolyte membranes.

| UPE-0.5 | Ionic conductivity | | | | |
| --- | --- | --- | --- | --- | --- |
| mS cm^−1^ | 30°C | 60°C | 90°C | 120°C | 150°C |
| 2.5 wt% | 0.01 | 0.068 | 0.234 | 0.347 | 0.588 |
| 5 wt% | 1.62×10^−3^ | 8.32×10^−3^ | 0.274 | 0.944 | 1.059 |
| 10 wt% | 0.042 | 0.195 | 0.234 | 0.549 | 1.247 |
| 20 wt% | 9.7×10^−3^ | 0.074 | 0.234 | 0.457 | 0.708 |
| UPE-1.5 | **Ionic conductivity** | | | | |
| 0 wt% | 1.62×10^−3^ | 0.017 | 0.165 | 0.554 | 0.684 |
| 2.5 wt% | 0.134 | 0.464 | 1.185 | 1.708 | 2.218 |
| 5 wt% | 1.06 | 1.28 | 1.769 | 3.71 | 5.76 |
| 10 wt% | 0.036 | 0.182 | 0.607 | 0.928 | 1.343 |
| 20 wt% | 0.0157 | 0.0864 | 0.319 | 0.75 | 1.417 |
| UPE-2.5 | **Ionic conductivity** | | | | |
| 2.5 wt% | 0.012 | 0.12 | 0.436 | 0.816 | 1.023 |
| 5 wt% | 0.457 | 0.88 | 1.258 | 1.866 | 2.754 |
| 10 wt% | 0.051 | 0.288 | 1.549 | 1.638 | 3.98 |
| 20 wt% | 0.02 | 0.074 | 0.617 | 1.483 | 2.138 |

**Figure S7.** DSC profiles of UPE with various LiTFSI concentrations.

**Figure S8.** XRD patterns of UPE with different varying LiTFSI content.

With the increase of the content of lithium salt, the crystallinity of the elastomer decreases, and the diffusive peak intensity decreases gradually.

**Figure S9.** Raman spectra of the UPE with different LiTFSI concentrations.

With the increase of Li^+^ concentration, the carbonyl peak (1730 cm^-1^) moves to a lower wave number, which means that the interaction between C=O and Li^+^ is enhanced, increasing the bond length of the C=O group.

**Figure S10.** LSV curves of UPE-1.5 with varying PEGDA contents at 120°C.

**Figure S11.** LSV curves of UPE-1.5 at different temperatures.

**
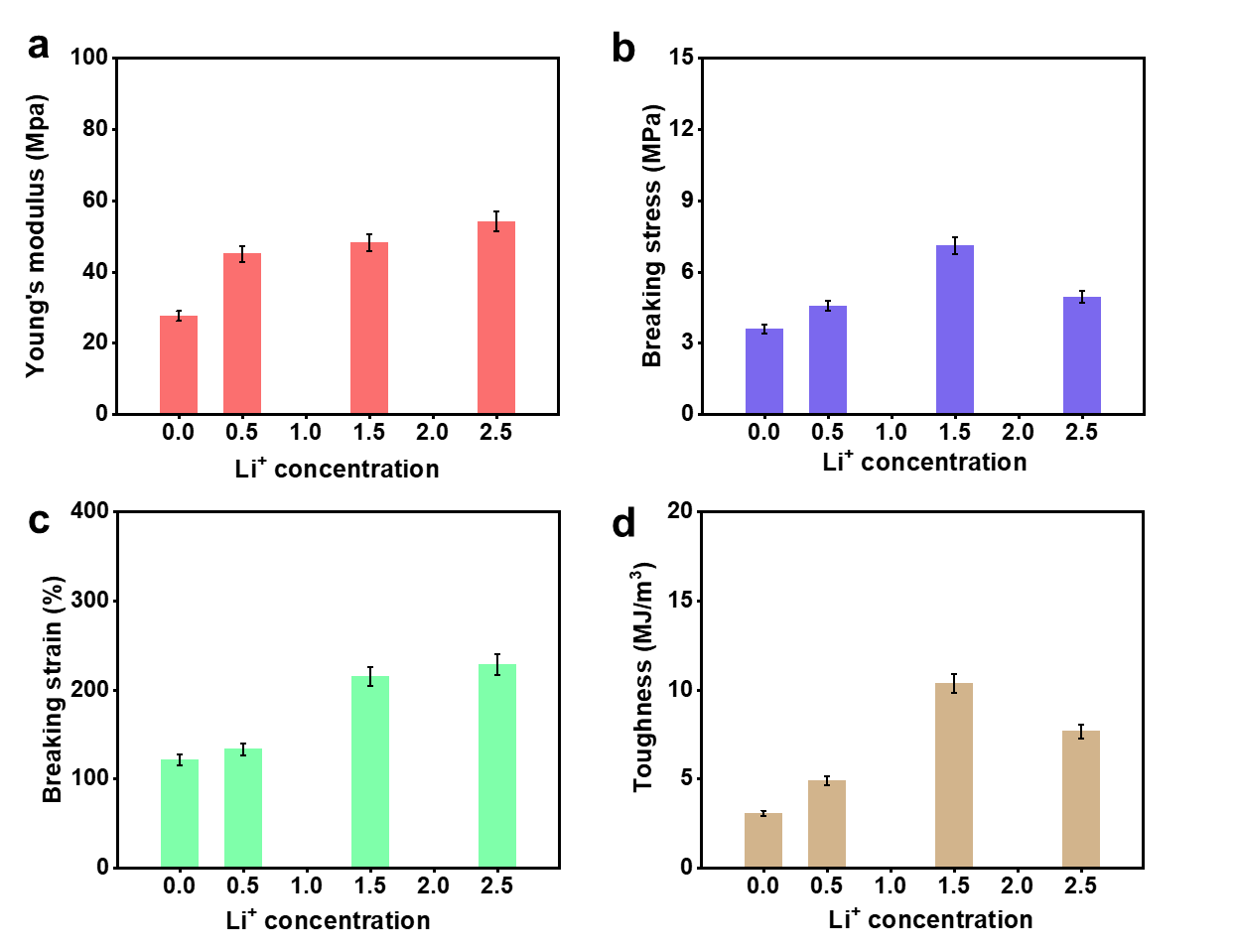
**

**Figure S12.** a) Summarized mechanical parameters of a) Young’s modulus, b) tensile strength, c) stretchability, and d) toughness with various LiTFSI concentrations.

As the concentration of LiTFSI increases, the formed Li^+^-carbonyl ion-dipole interaction will continuously strengthen the polymer network and slow down the relaxation kinetics of the polymer chains.


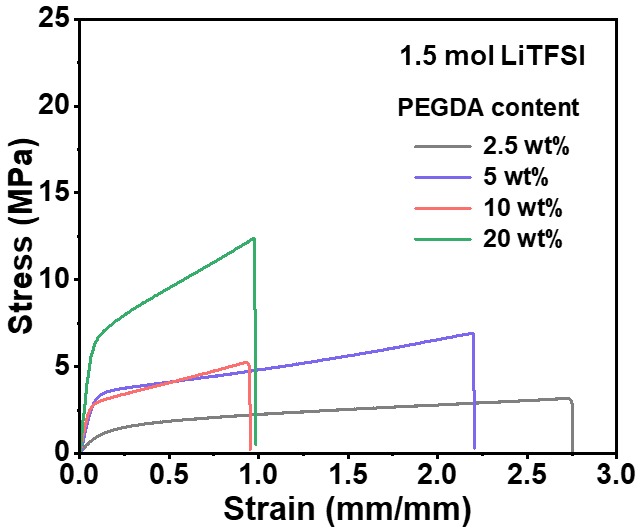


**Figure S13.** Stress-strain curves of UPE-1.5 with varying PEGDA contents.

**
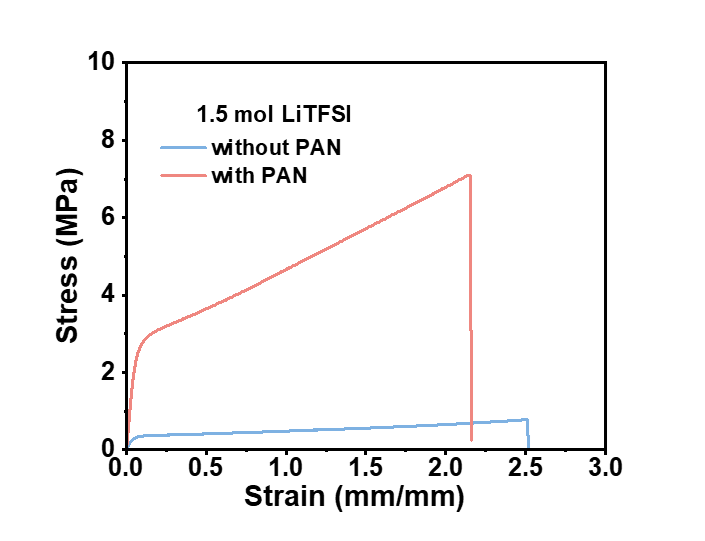
**

**Figure S14.** Stress-strain curves of UPE-1.5 with and without PAN fibers.

Figure S14 demonstrates that incorporating a PAN framework significantly enhances mechanical performance.

**
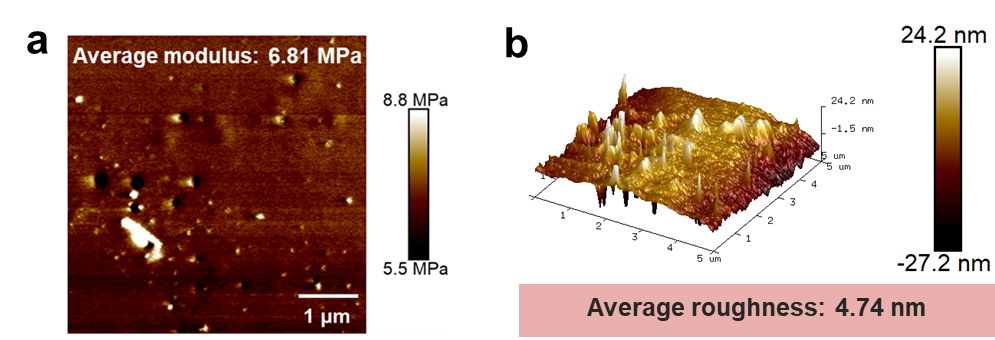
**

**Figure S15.** AFM scans of the UPE membrane surfaces, showing Young's modulus and average roughness of UPE-1.5 membrane.

Atomic force microscopy (AFM) analysis of the self-supporting film revealed an average surface roughness of around 4.74 nm, demonstrating the excellent component compatibility of the copolymer.


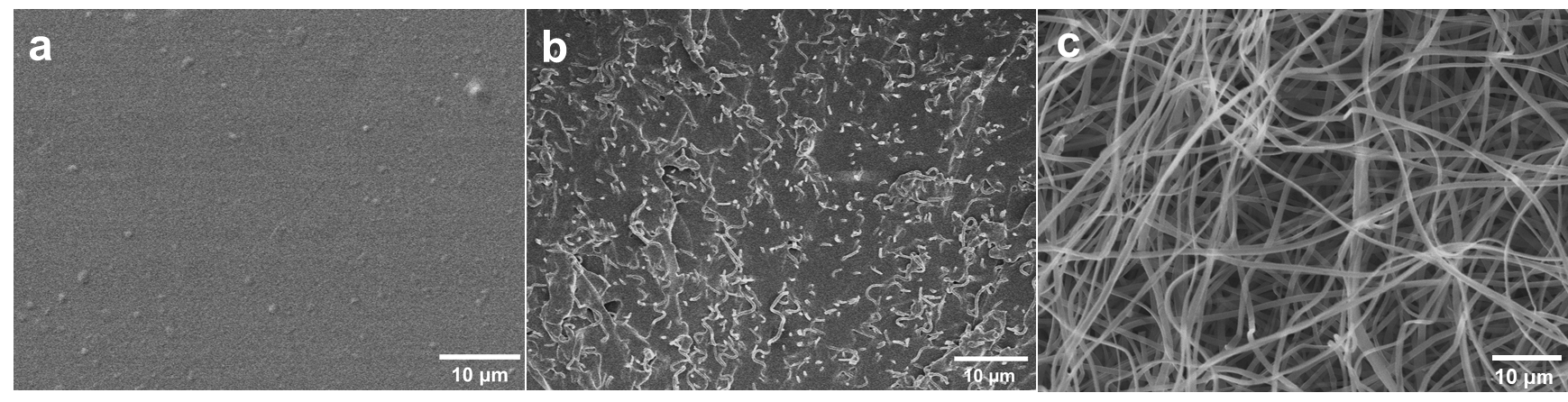


**Figure S16.** a-b) Top-surface and cross-sectional SEM images of the UPE-1.5 membrane. c) SEM image of the PAN fiber substrates.

**Figure S17.** TGA curves of UPE with various LiTFSI concentrations.

**
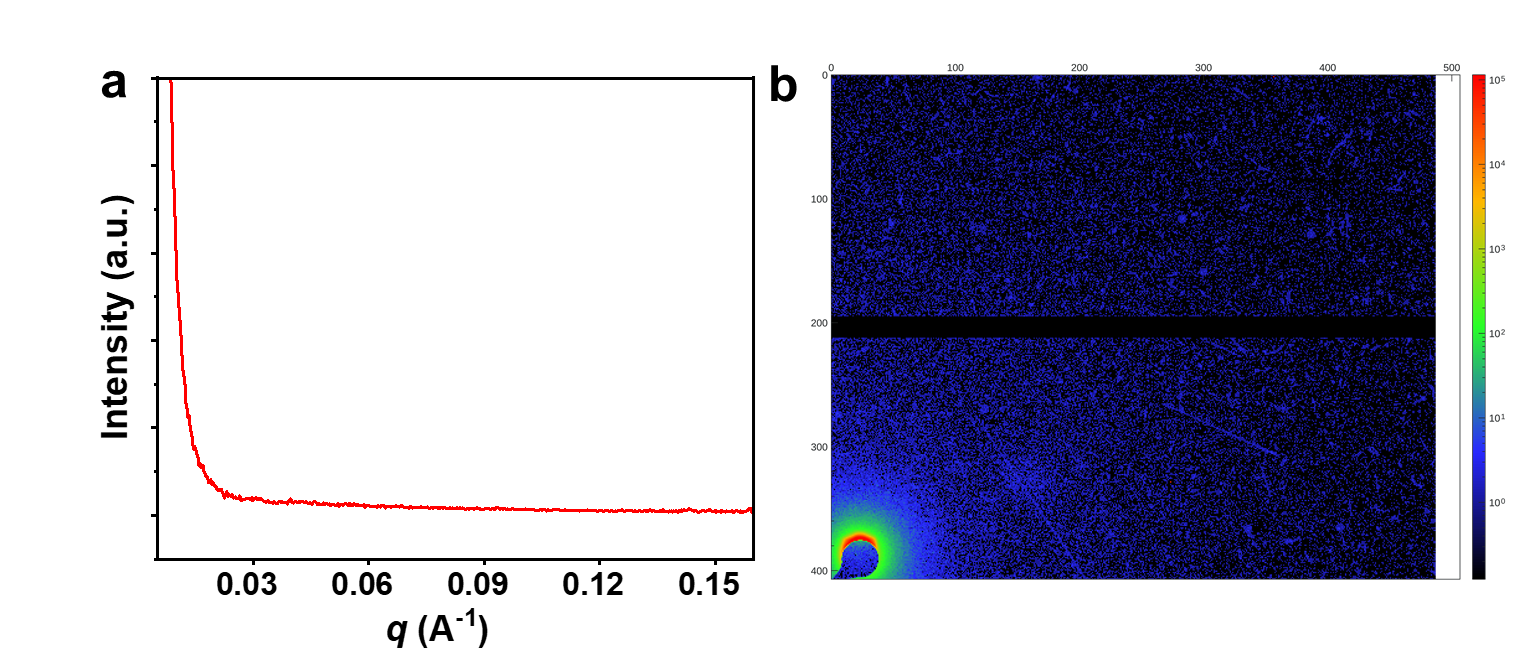
**

**Figure S18.** a) SAXS profile and b) 2D SAXS pattern of UPE-1.5 membrane.

Combined with previous experimental results, excessive lithium salts fail to continually increase ion-dipole numbers and intensities progressively. Instead, it disrupts established dynamic interactions, elevating internal resistance and narrowing the voltage window, ultimately resulting in concurrent degradation of both mechanical and electrochemical properties.

**Figure S19.** The ^7^Li NMR spectra of UPE-0.5 and UPE-1.5.


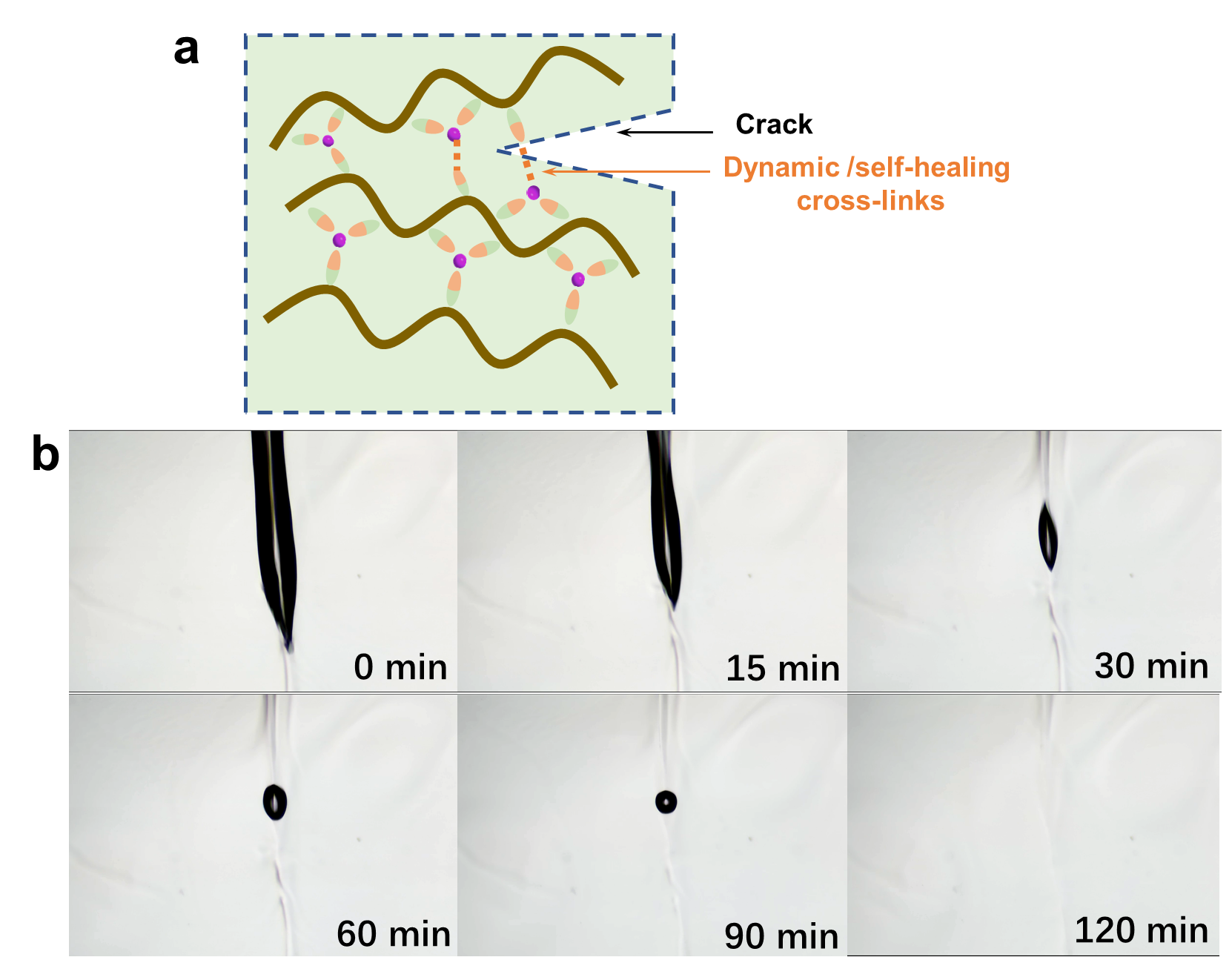


**Figure S20.** a) Schematic diagram of the self-healing mechanism inside UPE. b) Optical microscope images of the damaged sample and healed crack for UPE-1.5.


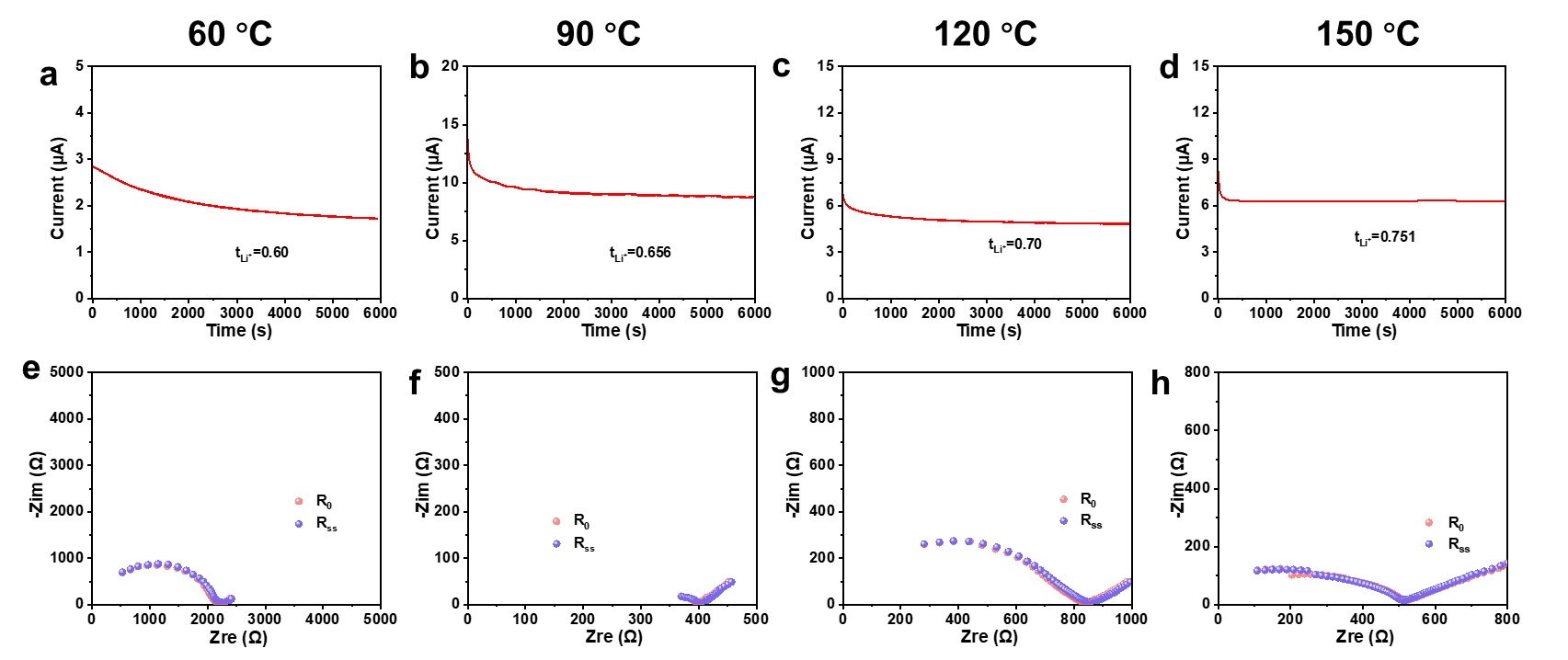


**Figure S21.** Chronoamperometry curves of Li|UPE-1.5|Li at a) 60 °C, b) 90 °C, c) 120 °C, and d) 150 °C. e-h) The corresponding alternating-current (AC) density impedance of the cell before and after polarization.

**Figure S22.** The ^19^F NMR Spectra of LiTFSI in different environments

**Figure S23.** a) The ^19^F NMR spectra of UPE-0.0 and UPE-1.5.


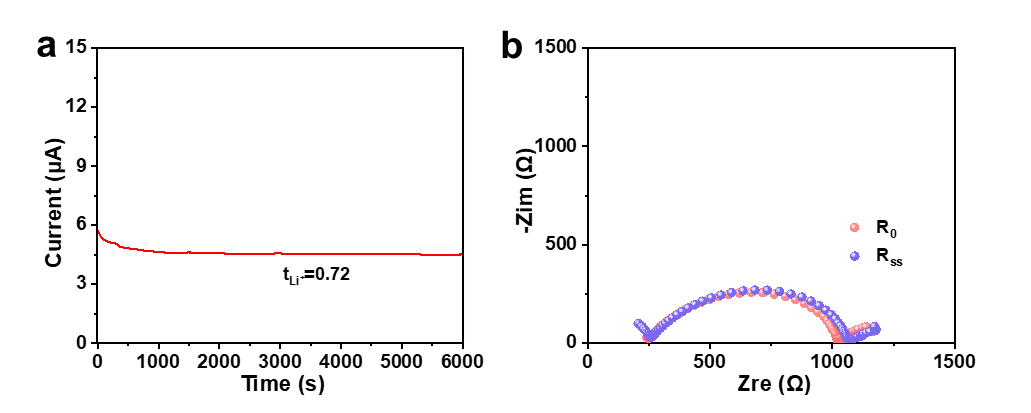


**Figure S24.** a) Chronoamperometry curves of Li|UPE-1.5|Li at 25°C. b) The corresponding alternating-current (AC) density impedance of the cell before and after polarization.

**Table S2.** The Li^+^ transference number at room temperature is mentioned in different literatures

|  | Reference | Li^+^ transference number |
| --- | --- | --- |
| ASSPE | Angew. Chem. Int. Ed. 2023, 62, e202306948 | 0.35 |
|  | Angew. Chem. Int. Ed. 2021, 60, 17701 | 0.23 |
|  | Adv. Mater. 2024, 36, 2403848 | 0.53 |
|  | Adv. Energy Mater. 2023, 13, 2203547 | 0.42 |
| QSSPE | Angew. Chem. Int. Ed. 2024, 63, e202406637 | 0.59 |
|  | Energy Environ. Sci., 2024,17, 2576-2587 | 0.48 |
|  | Angew. Chem. Int. Ed. 2023, 62, e202310006 | 0.55 |
|  | Adv. Mater. 2023, 35, 2304951 | 0.34 |


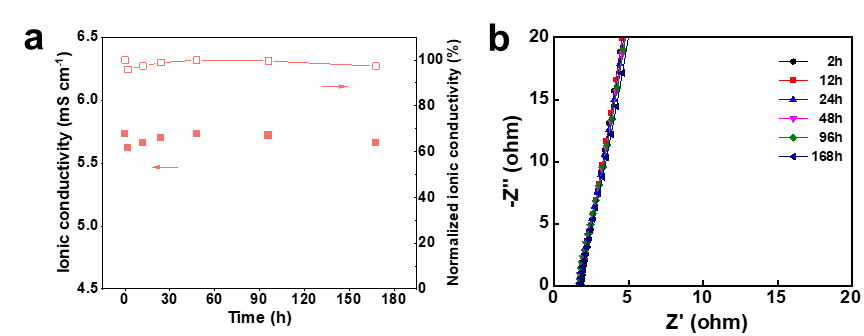


**Figure S25.** Change of ionic conductivity of UPE-1.5 at 150 ℃ during different storage times.


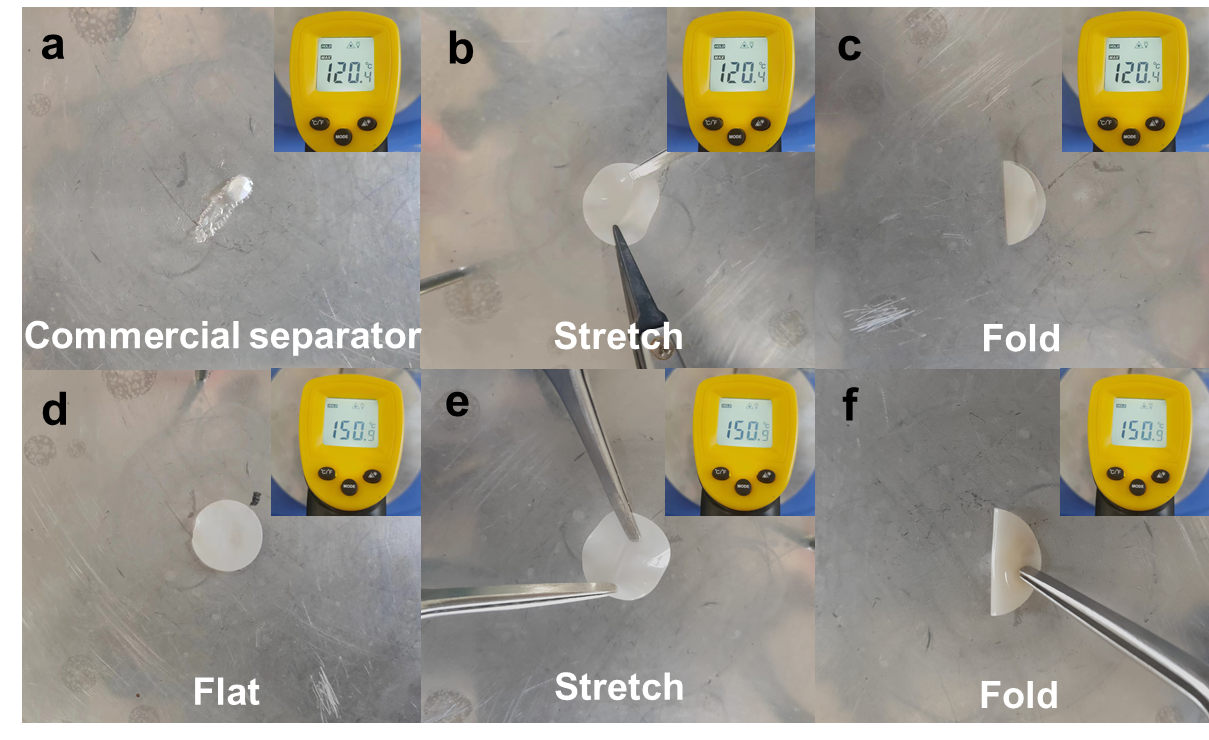


**Figure S26.** Digital photographs of the commercial separator and UPE-1.5 at 120 ℃ and 150 ℃. UPE can still maintain good flexibility and toughness, and there is no deterioration of the polymer film such as shrinkage and fracture at high temperatures.


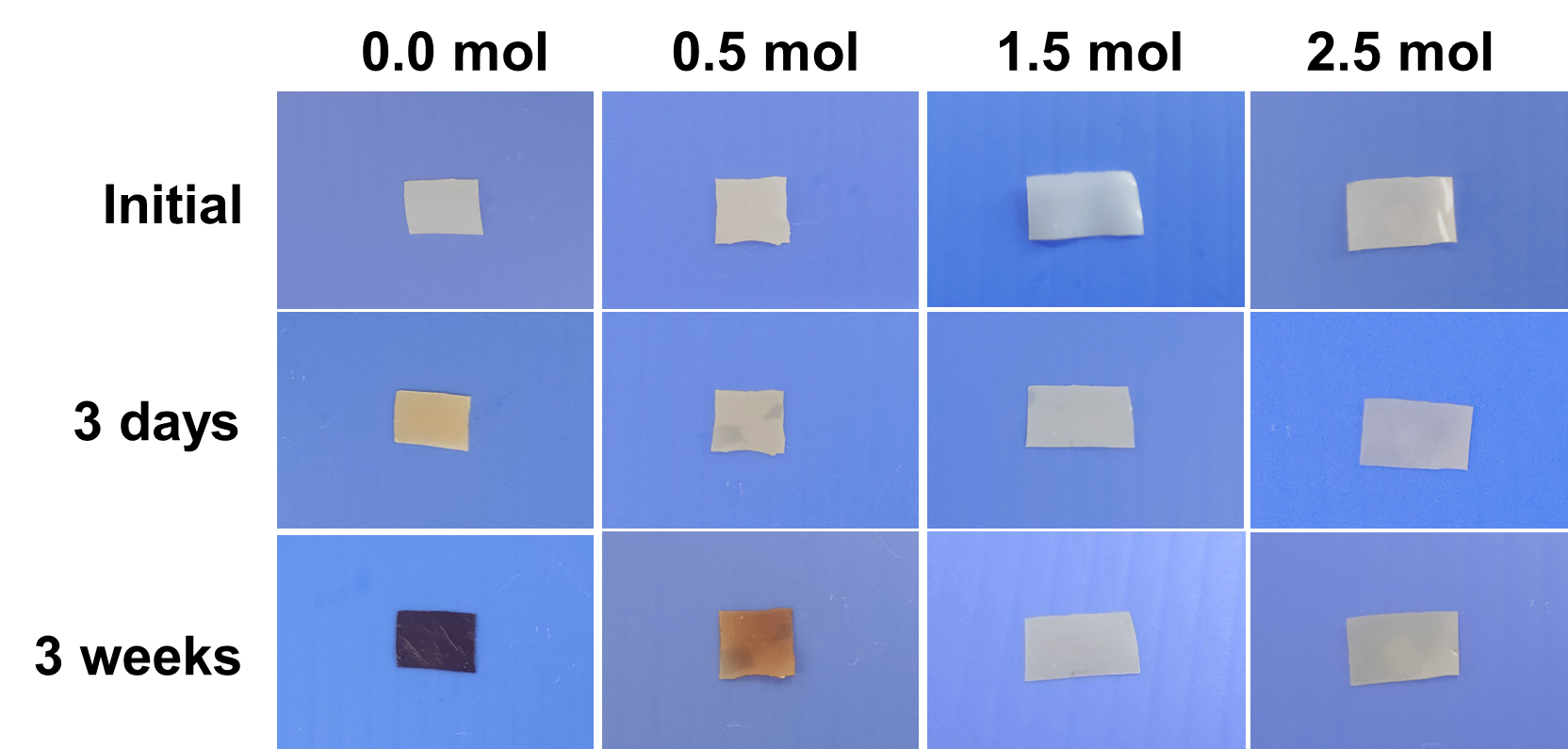


**Figure S27.** Digital images of UPE with different LiTFSI concentrations during different storage times.


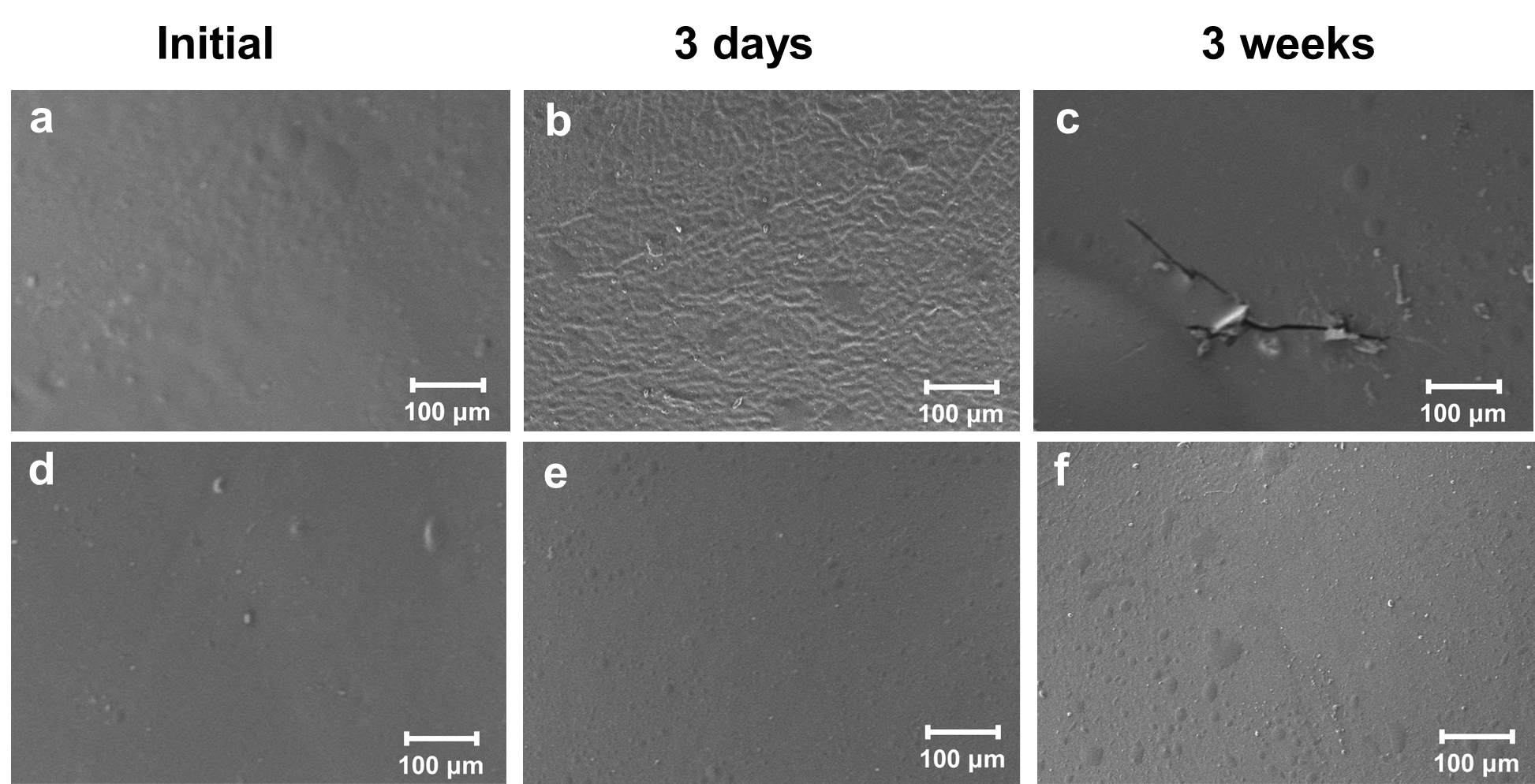


**Figure S28.** SEM images of UPE-0.5 membrane and UPE-1.5 membrane during different storage times.

**Figure S29.** Stress−strain curves of UPE-1.5 during different storage times.

**Figure S30.** Critical current density (CCD) of symmetric Li cell measured from 0.1 to 2.2 mA cm^−2^.

**Figure S31.** Cycling performance of Li symmetric cells with UPE-0.5, UPE-1.5, and UPE-2.5 at the current density and areal capacity of 0.2 mA cm^−2^ and 0.2 mAh cm^−2^ at 120 °C.

**Figure S32.** Long-term lithium plating/stripping experiment for Li||Li symmetric cell with UPE-1.5 at 25 °C at the current density and areal capacity of 0.1 mA cm^−2^ and 0.1 mAh cm^−2^.

**Figure S33.** Cycling performance of Li symmetric cells with UPE-1.5 at the current density and areal capacity of 0.5 mA cm^−2^ and 0.5 mAh cm^−2^ at 100 °C.


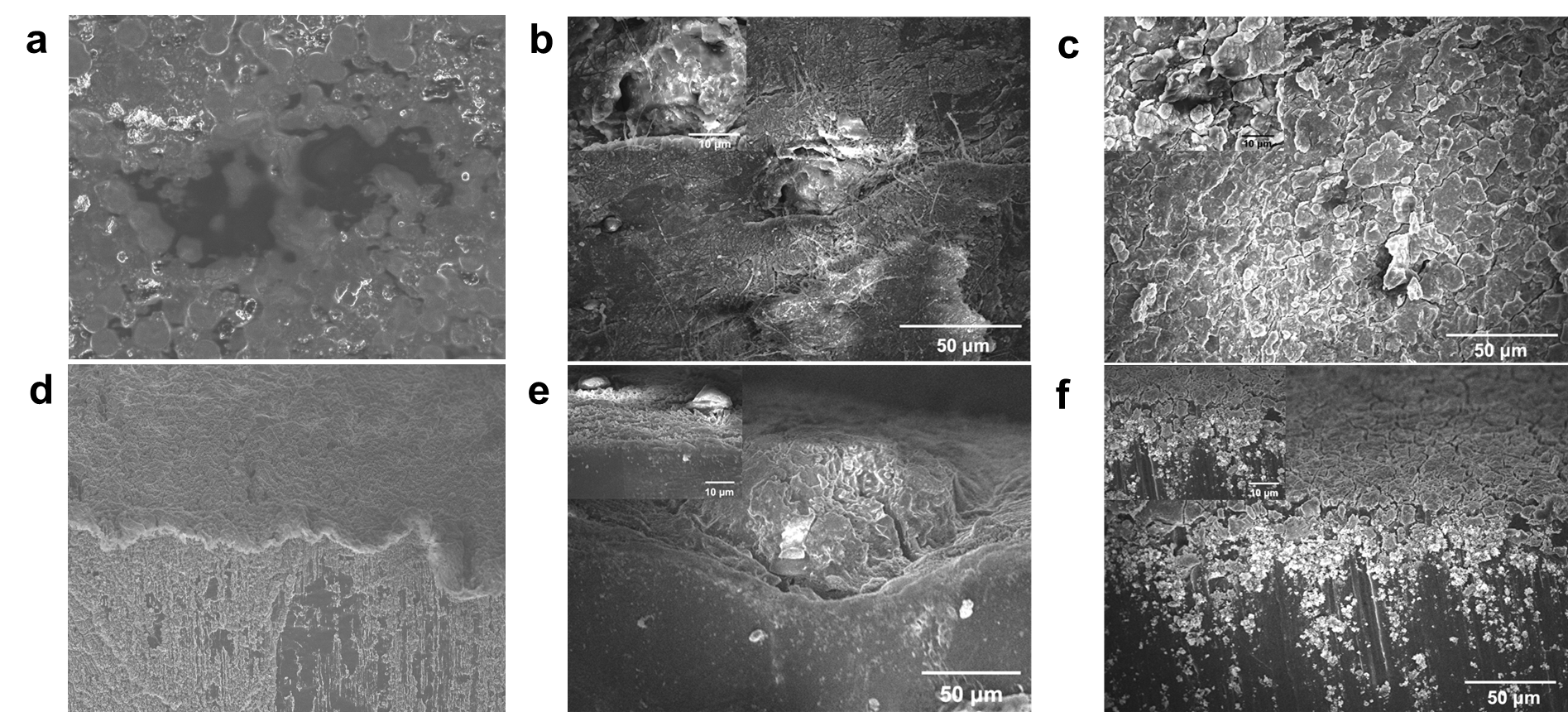


**Figure S34.** Top-surface and cross-sectional SEM images of the Li metal before and after Li plating-stripping cycles in symmetric cells using a, d) UPE-1.5, b, e) UPE-0.5, c, f) UPE-2.5.


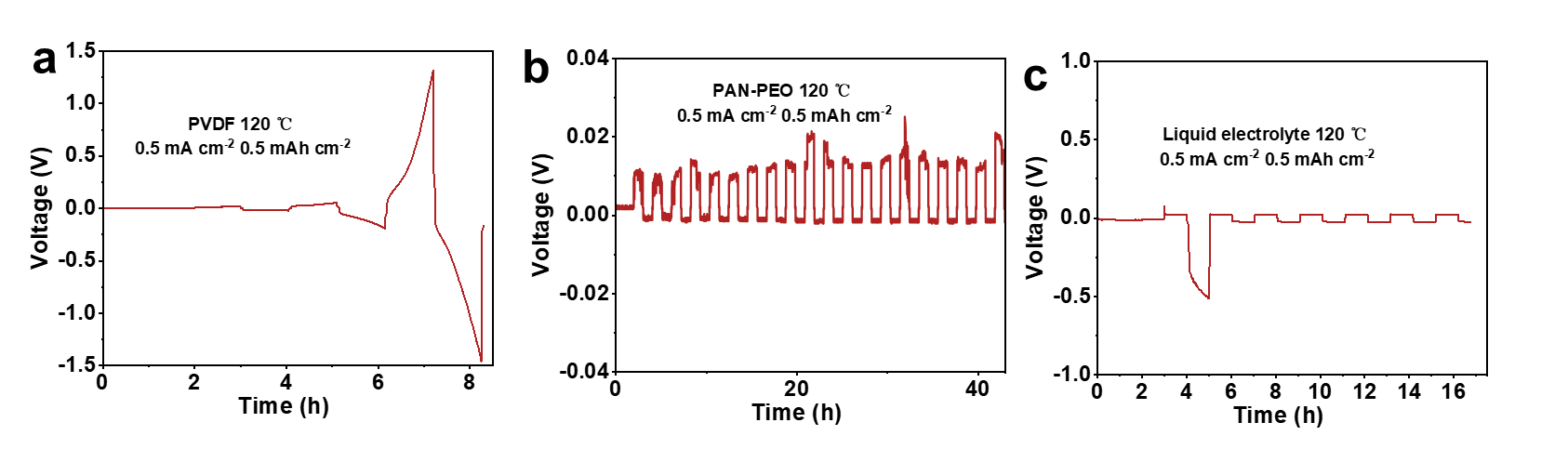


**Figure S35.** Cycling performance of Li symmetric cells with other electrolyte including a) PVDF, b) PEO with PAN fibers, c) commercial liquid electrolyte at the current density and areal capacity of 0.5 mA cm^−2^ and 0.5 mAh cm^−2^ at 120 °C.


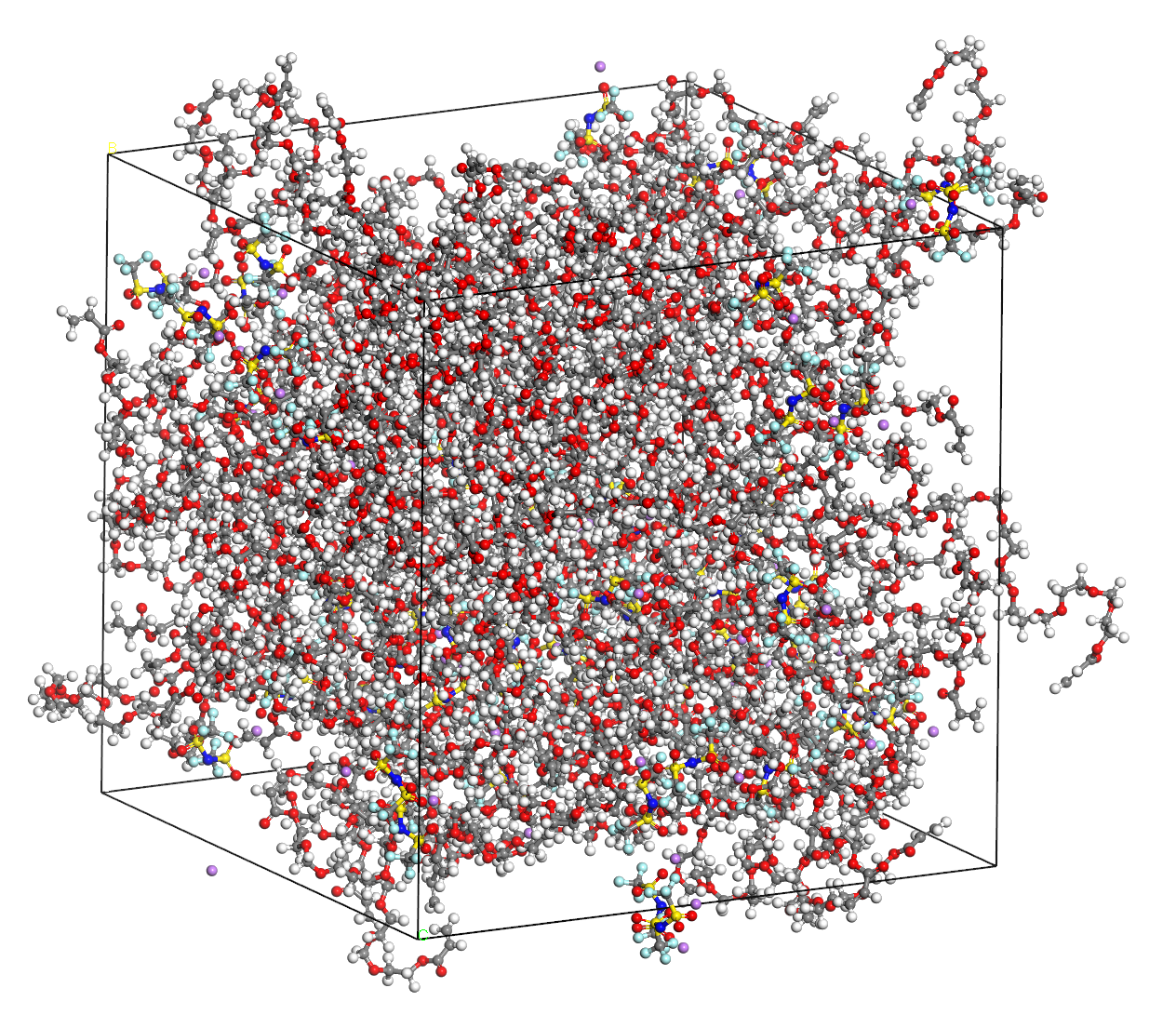


**Figure S36.** The simulation box of the simulation system for PEGDA at 150 °C


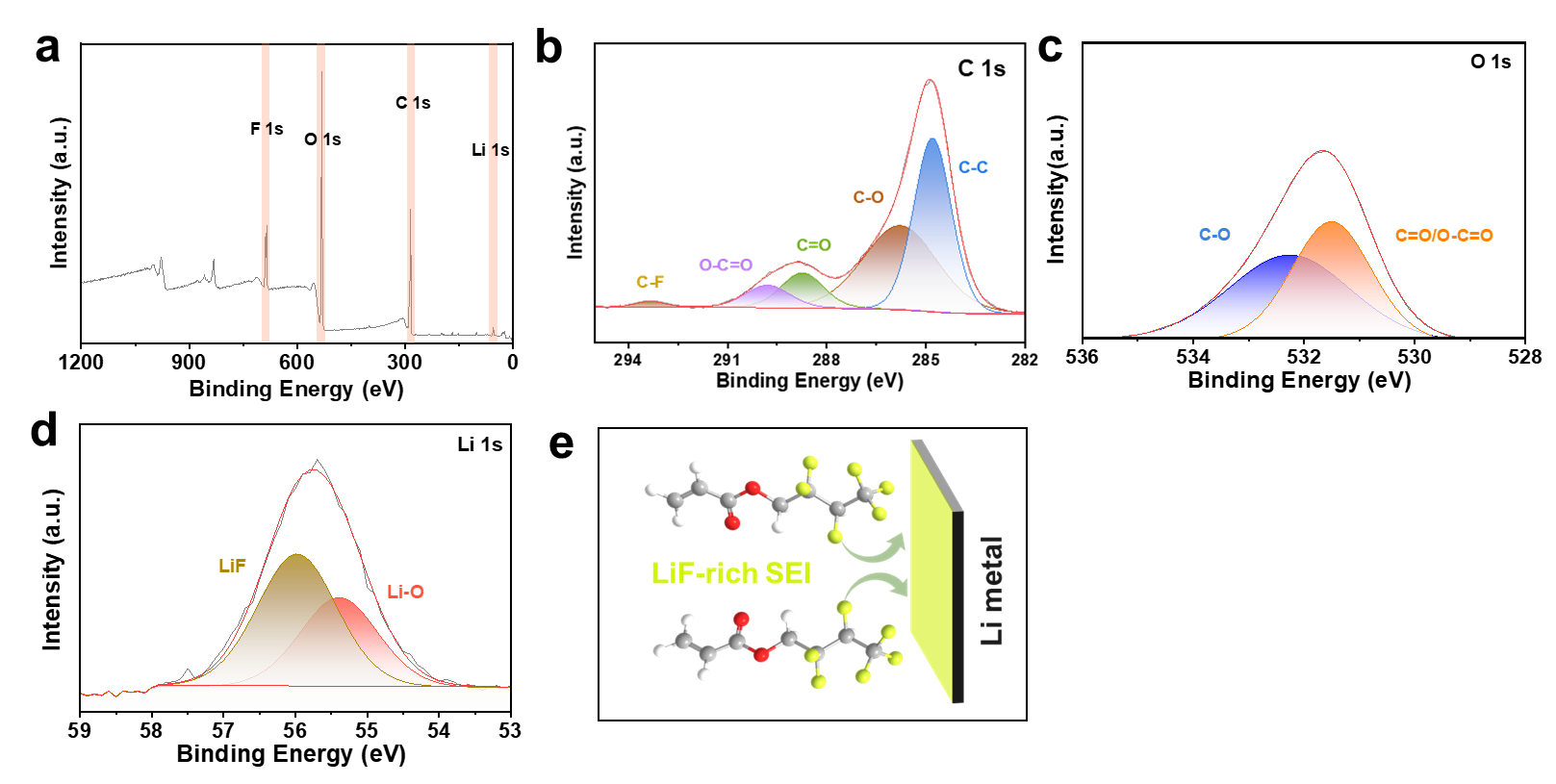


**Figure S37.** a) XPS survey spectra, b) C1s, c) O1s, d) Li1s XPS spectra of the SEI compositions of cycled symmetric Li cell with UPE-1.5. e) Schematic diagram of forming an LiF-rich interface layer on the surface of Li metal anode.

The assembled symmetric Li metal battery was cycled at 0.5 mA cm^−2^, 0.5 mAh cm^−2^ for 50 hours. Then the battery was disassembled in an Ar-filled glovebox. After rinsing the surface of the Li metal surface with TEGDME, XPS analysis was conducted.

**Figure S38.** CV curves of LFP|UPE-1.5|Li at 120 °C.

**Figure S39.** a) Cycling performance of LFP||Li with UPE-0.5 and UPE-2.5 at 1.0 C at 120 °C.


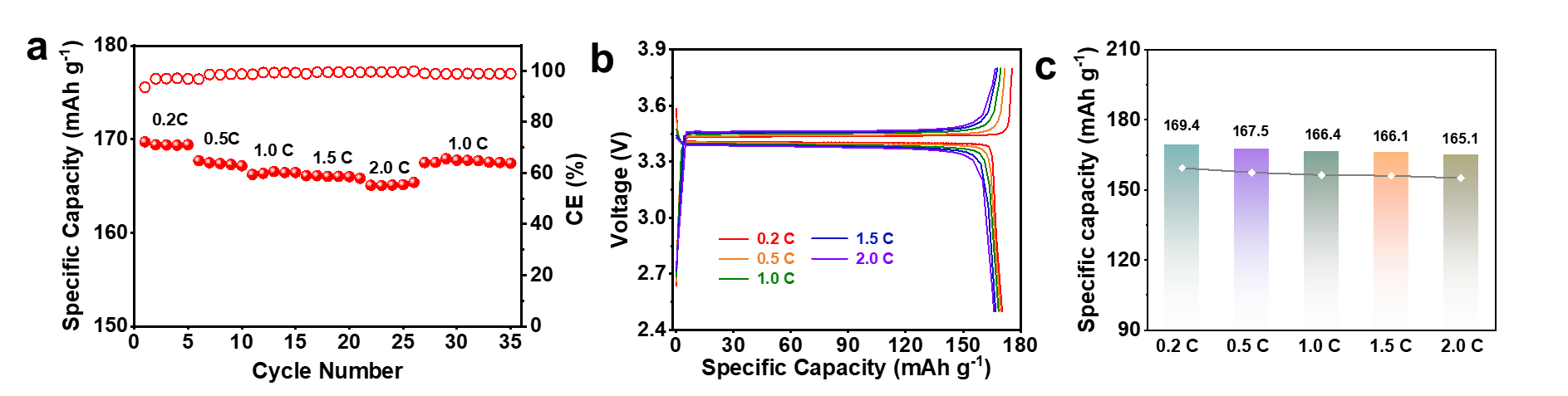


**Figure S40.** a) Rate performance and b) charge/discharge curves of LFP|UPE-1.5|Li batteries at 120 °C. c) The specific capacity shown at different rates.


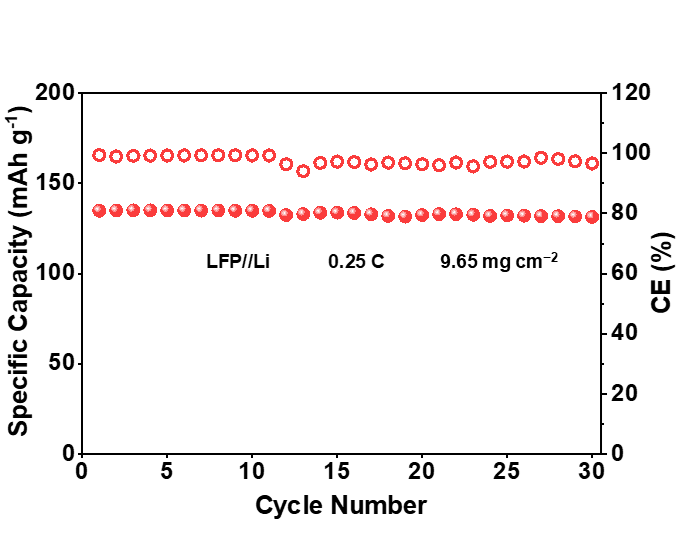


**Figure S41.** Cycling stability of high-loading LFP|UPE-1.5|Li at 0.25 C at 120 °C.


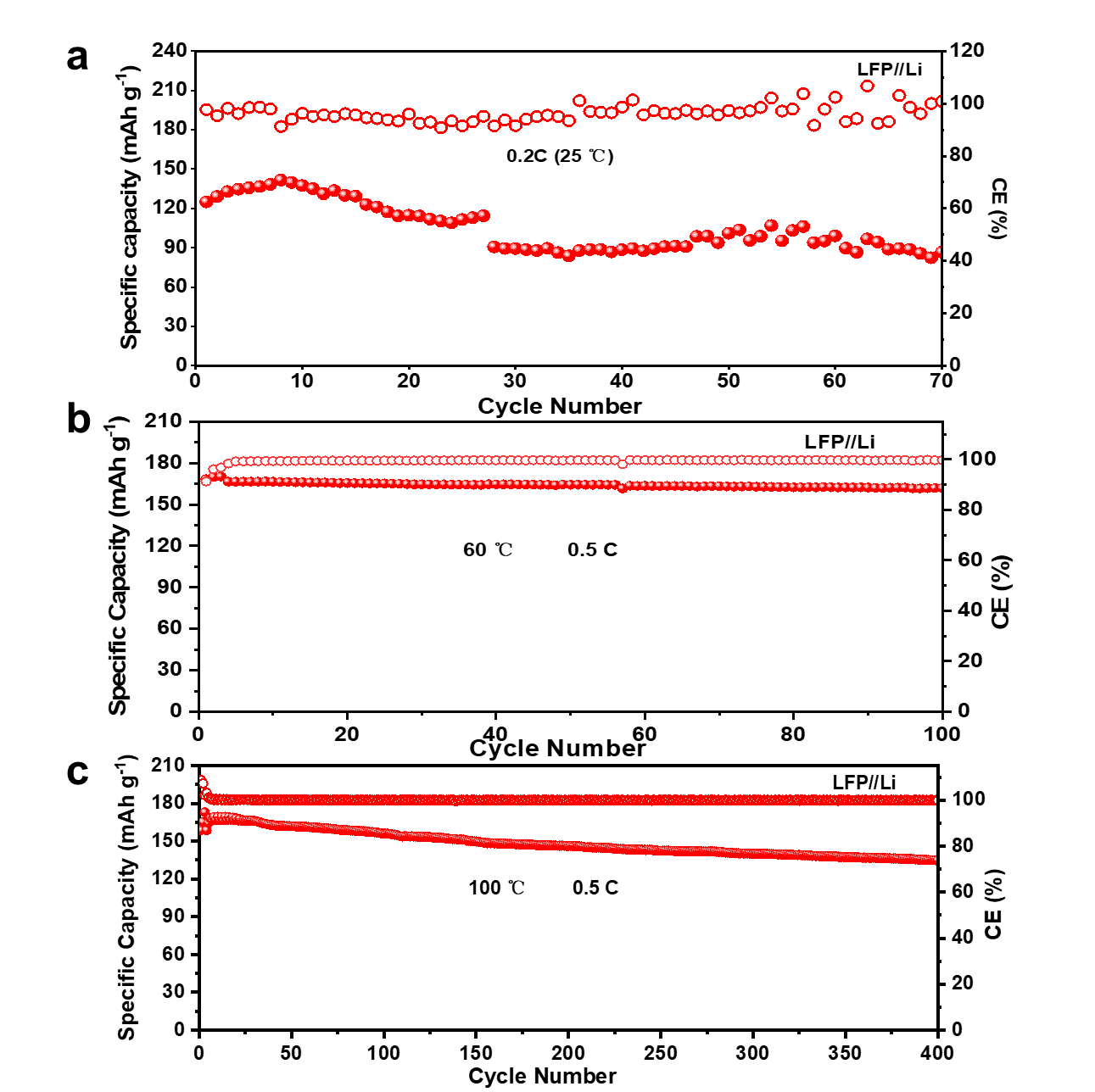


**Figure S42.** Capacity retention performance of LFP|UPE-1.5|Li at 25°C (0.2C), 60°C (0.5C), and 100°C (0.5C).


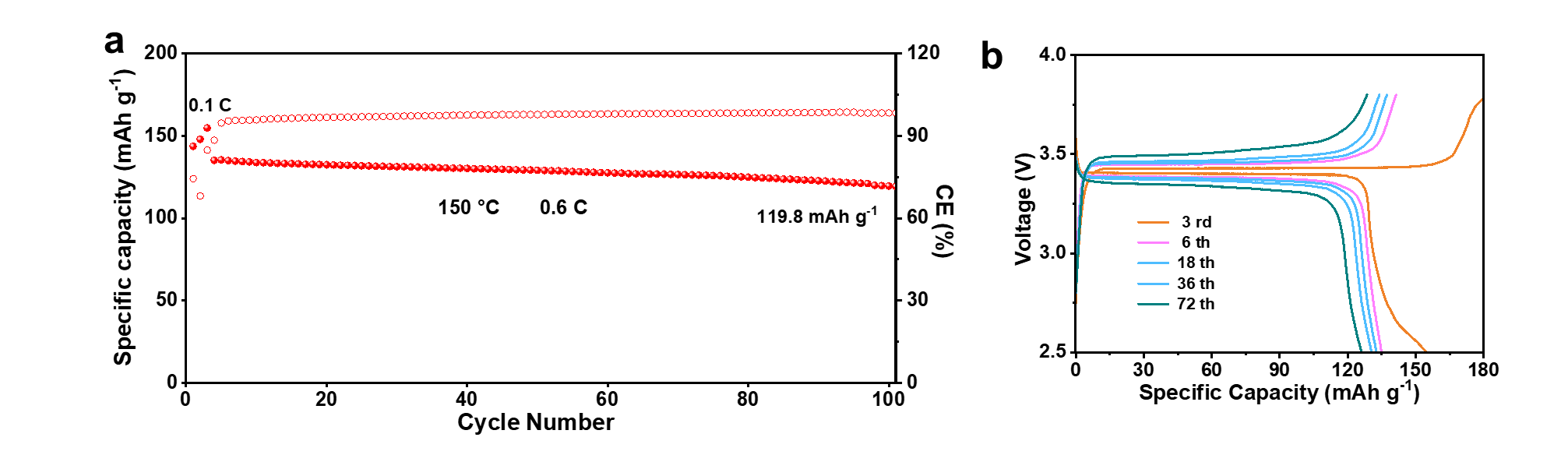


**Figure S43.** Capacity retention performance of LFP|UPE-1.5|Li at 0.6 C, cycled at 150 °C.

Table S3. Past reports on high-temperature cycle performance (>60 °C)

| Temperature | Cycling Number | References |
| --- | --- | --- |
| 100 | 50 | Angew. Chem. Int. Ed. 2022, 61, e202207645 |
| 80 | 280 | Energy Environ. Sci., 2025,18, 1696-1706 |
| 80 | 400 | J. Am. Chem. Soc. 2025, 147, 4089–4099 |
| 80 | 500 | Angew. Chem. Int. Ed. 2024, 63, e202410982 |
| 80 | 100 | Energy Storage Mater., 2019, 23, 646-652 |
| 70 | 65 | Nat. Energy, 2019, 4, 882–890 |
| 90 | 100 | Angew. Chem. Int. Ed. 2020, 59, 15109 |
| 100 | 40 | Chem. Sci., 2015,6, 6601-6606 |
| 80 | 300 | Adv. Energy Mater. 2022, 12, 2201631 |
| 120 | 160 | Adv. Eng. Mater., 2019, 21, 1900055 |
| 90 | 200 | Energy Storage Mater., 2019, 17, 309-316 |
| 70 | 50 | Nano Lett. 2019, 19, 3066−3073 |
| 100 | 200 | Adv. Energy Mater., 2020, 10, 1903441 |


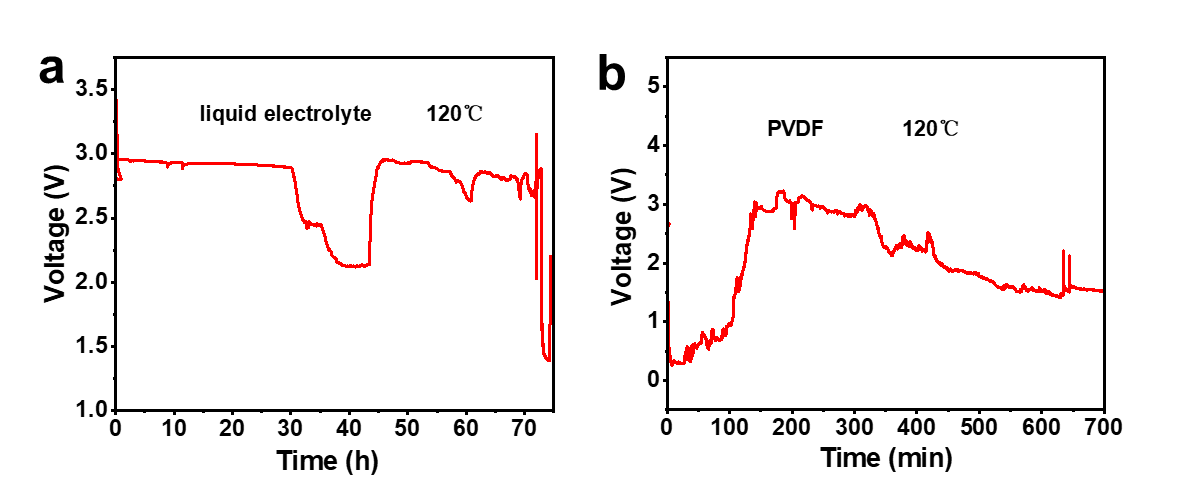


**Figure S44.** Charge and discharge curve of other electrolytes including a) liquid electrolyte and b) PVDF electrolyte at 120°C at 0.1C.

**Reference**

[1] K. Xu, Y. Lam, S. S. Zhang, T. R. Jow, T. B. Curtis, *The Journal of Physical Chemistry C* **2007**, *111*, 7411-7421.

[2] K. M. Abraham, Z. Jiang, B. Carroll, *Chemistry of Materials* **1997**, *9*, 1978-1988.

[3] U. Essmann, L. Perera, M. L. Berkowitz, T. Darden, H. Lee, L. G. Pedersen, *The Journal of Chemical Physics* **1995**, *103*, 8577-8593.

[4] H. A. Posch, W. G. Hoover, F. J. Vesely, *Physical Review A* **1986**, *33*, 4253-4265.

[5] M. Parrinello, A. Rahman, *Journal of Applied Physics* **1981**, *52*, 7182-7190.
